# Single Atom‐Doped Nanosonosensitizers for Mutually Optimized Sono/Chemo‐Nanodynamic Therapy of Triple Negative Breast Cancer

**DOI:** 10.1002/advs.202206244

**Published:** 2023-01-16

**Authors:** Qiqing Chen, Min Zhang, Hui Huang, Caihong Dong, Xinyue Dai, Guiying Feng, Ling Lin, Dandan Sun, Dayan Yang, Lin Xie, Yu Chen, Jia Guo, Xiangxiang Jing

**Affiliations:** ^1^ Department of Ultrasonography Hainan General Hospital/Hainan Affiliated Hospital of Hainan Medical University Haikou 570311 P. R. China; ^2^ Materdicine Lab School of Life Sciences Shanghai University Shanghai 200444 P. R. China; ^3^ Department of Ultrasound Zhongshan Hospital Fudan University and Shanghai Institute of Medical Imaging Shanghai 200032 P. R. China; ^4^ Shuguang Hospital Affiliated to Shanghai University of Traditional Chinese Medicine Shanghai 201203 P. R. China

**Keywords:** chemodynamic therapy, single‐atom catalysis, sonodynamic therapy, triple‐negative breast cancer therapy

## Abstract

Sonodynamic therapy (SDT) represents a promising therapeutic modality for treating breast cancer, which relies on the generation of abundant reactive oxygen species (ROS) to induce oxidative stress damage. However, mutant breast cancers, especially triple‐negative breast cancer (TNBC), have evolved to acquire specific antioxidant defense functions, significantly limiting the killing efficiency of SDT. Herein, the authors have engineered a distinct single copper atom‐doped titanium dioxide (Cu/TiO_2_) nanosonosensitizer with highly catalytic and sonosensitive activities for synergistic chemodynamic and sonodynamic treatment of TNBC. The single‐atom Cu is anchored on the most stable Ti vacancies of hollow TiO_2_ sonosensitizers, which not only substantially improved the catalytic activity of Cu‐mediated Fenton‐like reaction, but also considerably augmented the sonodynamic efficiency of TiO_2_ by facilitating the separation of electrons (e^−^) and holes (h^+^). Both the in vitro and in vivo studies demonstrate that the engineered single atom‐doped nanosonosensitizers effectively achieved the significantly inhibitory effect of TNBC, providing a therapeutic paradigm for non‐invasive and safe tumor elimination through the mutual process of sono/chemo‐nanodynamic therapy based on multifunctional single‐atom nanosonosensitizers.

## Graphical abstract

The Cu/TiO_2_‐PEG nanosonosensitizer with highly catalytic and sonosensitive activities are developed for synergistic chemodynamic and sonodynamic treatment of triple‐negative breast cancer. The single‐atom Cu doping not only improved the Cu‐mediated Fenton‐like catalytic activity, but also augmented the sonodynamic efficiency of TiO_2_ by facilitating the separation of electron‐hole pairs. This nanosystem provided a novel paradigm for the effective treatment of breast tumors.

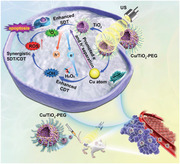

## 1 Introduction

As the most common cancer worldwide, breast cancer has evolved into one of the major public health problems threatening women's health,^[^ ^1^ ^]^ especially triple‐negative breast cancer (TNBC), the most aggressive tissue subtype,^[^ ^2^ ^]^ which is characterized by refractory, relapse‐prone, and low overall survival features.^[^ ^3^ ^]^ Chemotherapy is currently the mainstream strategy to inhibit the progression of TNBC, but it is limited by poor efficacy and severe side effects.^[^ ^4^ ^]^ It is highly urgent to develop high‐efficient and low‐toxic therapeutic protocols for suppressing TNBC.^[^ ^5^ ^]^ Fortunately, considerable technological success has been achieved in the field of cancer nanomedicine, which demonstrates great potential in clinical development and provides various alternatives for more effective cancer therapeutics.^[^ ^6^ ^]^

Of particular note is that ultrasound (US)‐responsive sonodynamic therapy (SDT), which employs US stimulation to activate the sonosensitizers in the tumor site for producing cytotoxic reactive oxygen species (ROS) and triggering tumor cells apoptosis and/or necrosis, represents an emerging modality for tumor treatment.^[^ ^7^ ^]^ The traditional organic sonosensitizers suffer from several critical drawbacks, such as high photonic cytotoxicity, low bioavailability, rapid metabolism, and undesirable therapeutic efficacy, impeding their further clinical translation.^[^ ^8^ ^]^ Compared with these organic sonosensitizers, the unique physicochemical properties and biological effects of inorganic nanosystems make them excellent alternatives as sonosensitizers. Titanium dioxide (TiO_2_) nanoparticles, as a typical class of inorganic sonosensitizers, have been extensively explored for sonodynamic tumor therapy,^[^ ^9^ ^]^ which can be activated by ultrasonic excitation to produce ROS through separating electrons (e^−^) and holes (h^+^) from the energy band structure, thus achieving the purpose of tumor killing. However, the US‐activated ROS generation efficacy of TiO_2_‐based sonosensitizers is still undesirable due to the inherently low quantum yields originating from the easy recombination of electrons (e^−^) and holes (h^+^). Recently, doping transitional metal ions has become an emerging strategy to heighten the photocatalytic activity of TiO_2_. When metal ions were doped into TiO_2_, the absorbance of TiO_2_ was significantly improved owing to the widening of surface plasmon absorption, and the Schottky barrier at the transitional metal ions‐TiO_2_ interface promoted the charge separation of photoinduced e^−^ and h^+^, thus enhancing its photocatalytic performance.^[^ ^10^ ^]^ By drawing the lessons from conventional photocatalysis, the doping of transitional metal ions into semiconductors could significantly enhance the separation efficiency of electrons (e^−^) and holes (h^+^) from the energy‐band structure upon external light irradiation. This principle is also applicable to transitional metal ion‐doped sonosensitizers, where the transitional metal ion doping could substantially augment the electrons (e^−^) and holes (h^+^) separation and inhibit their recombination, thus the production efficiency of ROS could be subsequently improved for inducing cancer cell death.^[^ ^11^ ^]^

Among numerous oxidative stress‐based nanotherapies, in addition to sonodynamic tumor therapy, Fenton catalytic production of ROS has contributed to the new concept of “nanocatalytic medicine”.^[^ ^12^ ^]^ The Fenton reaction‐based catalytic nanotherapy, typically utilizes ferrous ions (Fe^2+^) to convert hydrogen peroxide (H_2_O_2_) into hydroxyl radical (·OH) under acidic conditions with the aid of Fenton catalysts, displaying the peroxidase‐like activity (POD‐like activity).^[^ ^13^ ^]^ However, the Fe^2+^‐mediated Fenton reactions proceed effectively in highly acidic conditions (pH 2–4), which is generally limited by relatively low catalytic efficiency during tumor therapy. In contrast, the copper‐based Fenton‐like nanocatalysts have proved to be a potential candidate for CDT due to their high efficiency in weakly acidic TME^[^ ^14^ ^]^ Therefore, it is highly desirable and necessary to design iron‐free nanoformulations containing copper, which have high therapeutic specificity and favorable catalytic performance and can be used for effective cancer treatment. Single‐atom catalysts are emerging as high‐performance nanocatalysts,^[^ ^15^ ^]^ which are attributed to the fully exposed atomic active metal sites, providing nearly 100% atomic dispersion and maximizing metal utilization, and finally exerting extraordinarily high catalytic activity.^[^ ^16^ ^]^ It has been demonstrated that the PEGylated single‐atom iron‐containing nanocatalysts could efficiently trigger in situ tumor‐specific Fenton reaction under the tumor microenvironment (TME) and selectively generate abundant toxic ·OH.^[^ ^17^ ^]^ The highly efficient catalytic reactions based on the unique microenvironment of tumors have laid a solid foundation for tumor‐specific catalytic therapeutics.

**Scheme 1:**
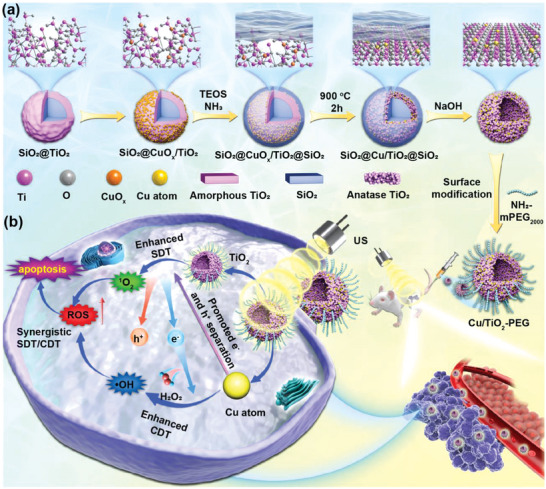
Schematic illustration for the construction of single copper atom‐doped titanium dioxide nanosonosensitizers and synergistic inhibitory mechanism of sono/chemo‐nanodynamic therapies for TNBC. a) Schematic diagram of the synthetic process of single‐atom Cu/TiO_2_‐PEG nanosonosensitizers by a reformative wrap‐bake‐strip method. b) Synergetic‐enhanced ROS generation of single‐atom Cu/TiO_2_‐PEG nanosonosensitizers for sono/chemo‐dynamic therapeutic process under US irradiation against TNBC.

Herein, we present the rational design and construction of single‐atom copper (Cu)‐doped hollow TiO_2_ nanosonosensitizers (Cu/TiO_2_) for synergistically enhanced sonodynamic and chemodynamic nanotherapies against TNBC. This single‐atom nanocatalyst‐based Cu/TiO_2_ nanosonosensitizer is fabricated by exclusively occupying well‐dispersed Cu atoms in the stable Ti vacancies of hollow TiO_2_ nanoparticles, which performs an atomic‐level and cooperative SDT and chemodynamic therapy (CDT) effects upon US activation (**Scheme** **1**). Concretely, the single‐atom Cu doping effectively promotes the separation efficiency of electrons (e^−^) and holes (h^+^) upon the US irradiation, thus guaranteeing a higher ROS yield than that of pure TiO_2_ nanosensitizers. In addition, the hollow TiO_2_ nanoparticles dispersed with single‐atom Cu possess Fenton catalytic active centers, resulting in favorable CDT performance for TNBC treatment. This single atom Cu/TiO_2_‐mediated synergistically augmented nanodynamic therapy (CDT/SDT) has been verified to substantially inhibit the growth of TNBC in a highly biosafe manner, providing a new paradigm for the effective and thorough treatment of refractory breast tumors.

## 2 Results and Discussion

### 2.1 Synthesis and Characterization of Single‐Atom Hollow Cu/TiO_2_ Nanosonosensitizers

The single‐atom hollow Cu/TiO_2_ nanosonosensitizers were fabricated by a reformative wrap‐bake‐strip method.^[^ ^18^ ^]^ Initially, the TiO_2_ coating was fabricated on the SiO_2_ nanoparticles, and the metal Cu precursor was adsorbed onto the TiO_2_ layer, then coated with silica to form core/shell‐structured (SiO_2_@CuO_x_/TiO_2_@SiO_2_). Second, the redistribution of metal atoms was confined by 900 °C calcination to form SiO_2_@Cu/TiO_2_@SiO_2_. Finally, the calcined SiO_2_@Cu/TiO_2_@SiO_2_ nanoparticles were dispersed into the alkaline solution to etch the SiO_2_ template and subsequently form the hollow Cu/TiO_2_ nanosonosensitizers (Scheme 1a). The Cu content in Cu/TiO_2_ was 7.06%, which was measured by inductively coupled plasma optical emission spectrometer (ICP‐OES). The step‐by‐step structural evolution during the synthesis process of Cu/TiO_2_ nanoparticles was revealed and demonstrated in Figure S1 (Supporting Information).

**Figure 1:**
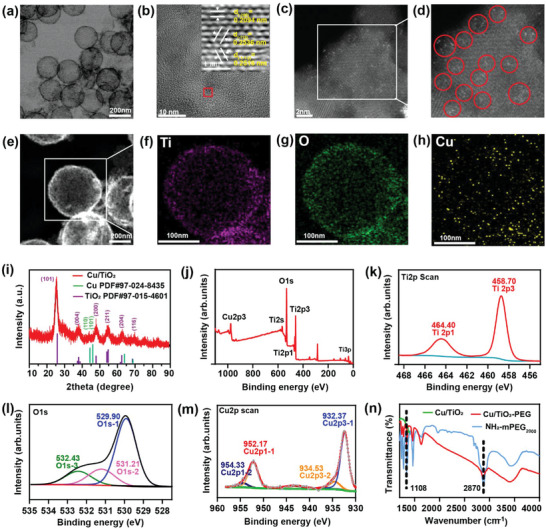
Characterization of single‐atom Cu/TiO_2_ nanosonosensitizers. a) TEM, b) HRTEM, and c,d) HAADF‐STEM images of single‐atom Cu/TiO_2_ nanosonosensitizers. e–h) STEM images e) and elemental mappings of Cu/TiO_2_ nanosonosensitizers, including f) Ti, g) O, and h) Cu elements. i) XRD patterns of Cu/TiO_2_ nanosonosensitizers. j) XPS spectrum of Cu/TiO_2_ and XPS spectrum of Cu/TiO_2_ in (k) Ti2p region, l) O1s region and m) Cu2p region. n) Fourier transform infrared (FTIR) spectra of Cu/TiO_2_, Cu/TiO_2_‐PEG, and NH_2_‐mPEG_2000_ from the range of 1000 to 4000 cm^−1^.

The transmission electron microscopy (TEM) and high‐resolution TEM (HRTEM) images exhibited that the well‐dispersed Cu/TiO_2_ nanosonosensitizers were composed of small TiO_2_ nanocrystals, and the attached TiO_2_ nanoparticles were highly crystallized (**Figure** **1**a,b). Lattice stripes with the plane spacing of 0.2094 nm, 0.2536 nm, and 0.2516 nm corresponded to (‐200), (‐112), and (‐1‐1‐2) lattice planes of TiO_2_, respectively, which confirmed the generation of the TiO_2_ nanoparticles. Besides, the high‐angle annular dark field scanning transmission electron microscopic (HAADF‐STEM) imaging with aberration correction was performed to determine the Cu single‐atoms fixed on Ti vacancies in the hollow TiO_2_ nanoparticles. Individually dispersed single Cu atoms were identified as the bright spots in the HAADF‐STEM images, as shown by the red marker, which also verifies the non‐aggregated state of the Cu single atoms (Figure 1c,d). We further conducted a HAADF‐STEM analysis on the Cu/TiO_2_ nanosonosensitizers, and directly imaged the local structure of isolated Cu atoms. The bright contrast spots (Cu atoms) were only located on the Ti atomic row, confirming that Cu atoms only existed in the Ti vacancies (Figure S2, Supporting Information). No other Cu configurations were detected. In addition, the energy dispersive X‐ray spectroscopy (EDS) and the element distribution mappings of Ti, O, and Cu elements were confirmed throughout the structure of Cu/TiO_2_ nanosonosensitizers (Figure 1e–h, Figure S3, Supporting Information).

The X‐ray diffraction (XRD) pattern certificated that only the diffraction signals from TiO_2_ could be distinguished (Figure 1i; Figure S4, Supporting Information). There were no crystal‐phase Cu‐based nanoparticle signals, which indirectly indicated that only Cu single‐atoms were highly isolated and dispersed in hollow TiO_2_ nanoparticles. The chemical state of individual elements in Cu/TiO_2_ nanoparticles was analyzed by X‐ray photoelectron spectroscopy (XPS) (Figure 1j). The obvious Ti and Cu signals in XPS indicated the presence of Cu single‐atoms within TiO_2_ nanoparticles. For the engineered hollow Cu/TiO_2_ nanosonosensitizers, the characteristic peaks of TiO_2_ at 485.70/464.40 eV and 529.90/531.21/532.43 eV could be observed in the Ti2p and O1s region of hollow Cu/TiO_2_ nanoparticles, respectively (Figure 1k,l).^[^ ^19^ ^]^ As shown in Figure 1m, the obvious Cu signal in XPS indicated the successful integration of Cu single‐atoms into TiO_2_ nanoparticles, in which the fitting peaks ≈932.37, 952.17, 934.53, 954.33, and 943.22 eV were assigned to Cu2p3/1, Cu2p1/1, Cu2p3/2, Cu2p1/2, and Cu sat, respectively.^[^ ^20^ ^]^

For enhancing the stability and biosafety of hollow Cu/TiO_2_ nanosonosensitizers under physiological conditions, the biocompatible methoxypolyethylene glycol amine 2000 (NH_2_‐mPEG_2000_) was grafted onto the surface of these Cu/TiO_2_ nanosonosensitizers (indicated as Cu/TiO_2_‐PEG). Fourier transform infrared (FTIR) spectrum demonstrated that NH_2_‐mPEG_2000_ was successfully modified onto Cu/TiO_2_ nanosonosensitizers. Generally, the peaks at 2870 cm^−1^ (the C—H stretching vibration) and 1108 cm^−1^ (the C—O—C stretching vibration) were the characteristic peaks of the PEG component (Figure 1n). The variation of particle size and zeta potential based on the typical dynamic light scattering (DLS) indicated the successful synthesis and surface modification of Cu/TiO_2_‐PEG (Figure S5, Supporting Information). The final hydrodynamic particle size of Cu/TiO_2_‐PEG was determined from 200.9 nm to 234.2 nm (Figure S5a, Supporting Information), and the zeta potential changed from −27.3 to −12.8 mV (Figure S5b, Supporting Information). In addition, after NH_2_‐mPEG_2000_ modification, the Cu/TiO_2_‐PEG nanoparticles exhibited desirable stability in the physiological solutions, such as water and fetal bovine serum (FBS), over 5 days, ensuring their further applications in the subsequent cellular and in vivo experiments (Figure S6, Supporting Information).

### 2.2 In Vitro Reactive Oxygen Species (ROS) Generation Upon US Activation and its Mechanism

**Figure 2:**
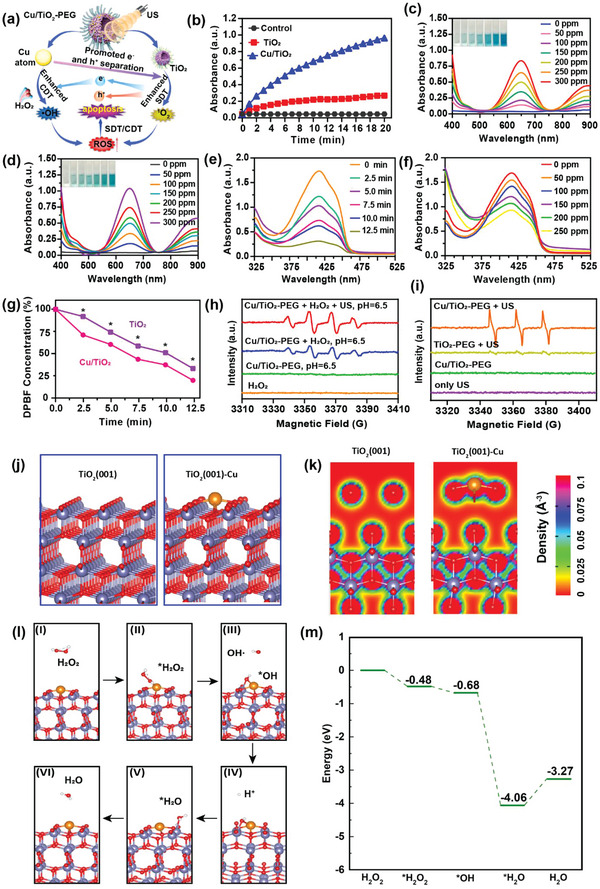
In vitro sono/chemo‐dynamic performance of single‐atom Cu/TiO_2_ nanosonosensitizers and density functional theory calculations. a) Schematic illustration of synergistically enhanced ROS generation of single‐atom Cu/TiO_2_ nanosonosensitizers under US irradiation. b) Time‐course absorbance of TMB to assess the ·OH generation capability of TiO_2_ and Cu/TiO_2_ through UV–vis measurement. c,d) UV–vis absorbance of TMB to evaluate the ·OH generation of Cu/TiO_2_ without c) or with d) US irradiation at different concentrations (0, 50, 100, 150, 200, 250, and 300 ppm). e) UV–vis absorbance of Cu/TiO_2_ containing DPBF exposure to US irradiation for different durations (0, 2.5, 5, 7.5, 10, and 12.5 min). f) UV–vis absorbance of Cu/TiO_2_ containing DPBF exposure to US irradiation (power density: 1.0 W cm^−2^, duty cycle: 50%, 5 min) at different concentrations (0, 50, 100, 150, 200, and 250 ppm). g) Relative absorption of DPBF after co‐incubation with Cu/TiO_2_ or TiO_2_ under US irradiation for different durations. h) BMPO and i) TEMP spin‐trapping ESR spectra of Cu/TiO_2_ under different conditions. j) Schematics and k) charge densities of pure TiO_2_(001) facet and Cu‐doped TiO_2_(001) facet (TiO_2_(001)‐Cu). l) The POD‐like activity catalytic mechanism and m) the energy diagram of the Cu‐doped TiO_2_(001) surface. The purple, orange, red, and white balls indicate the Ti, Cu, O, and H atoms, respectively. *P< 0.05, **P< 0.01, ***P<0.001, ****P<0.0001 and ns for non‐significant.

**Figure** **2**a displayed the schematic illustration of sono/chemo‐dynamic activities of single‐atom Cu/TiO_2_ under US irradiation in vitro. Cu single atom could convert H_2_O_2_ into highly toxic ·OH, displaying POD‐like activity under acidic conditions. 3,3′,5,5′‐tetramethyl‐benzidine (TMB) was applied to evaluate the production of ·OH according to its color changes. The UV–vis absorbance results demonstrated the time‐ and concentration‐dependent manners of the ·OH generation induced by Cu/TiO_2_ (Figure 2b,c), due to the single atom Cu‐mediated Fenton‐like catalytic reaction. More importantly, the UV–vis absorbance of TMB increased significantly after US irradiation (power density: 1.0 W cm^−2^, duty cycle: 50%, 5 min), illustrating that US irradiation‐triggered cavitation effect could accelerate the mass transfer and chemical reaction rates of the Fenton‐like reaction ^[^ ^21^ ^]^ and thus improve the production efficiency of ·OH to augment CDT (Figure 2d; Figure S7, Supporting Information). In addition, 1,3‐diphenylisobenzofuran (DPBF), as the singlet oxygen (^1^O_2_) probe, was used to assess the sonodynamic effect of the engineered single‐atom Cu/TiO_2_ nanosonosensitizers. As shown in Figure 2e,f, the content of ^1^O_2_ generated by Cu/TiO_2_ also exhibited time and concentration dependence, respectively. The quantitative decrease of DPBF absorption intensity at 416 nm under US irradiation was further measured in the presence of Cu/TiO_2_ or pure TiO_2_, respectively (Figure 2g). The ^1^O_2_ production efficiency of Cu/TiO_2_ nanosonosensitizers was obviously higher than that of pure TiO_2_, indicating that the presence of Cu single atoms in Cu/TiO_2_ nanosonosensitizers could improve the ^1^O_2_ production and subsequent SDT performance. This may be attributed to the fact that Cu single‐atom significantly improved the US excited electron transfer efficiency, which elevated the separation efficiency of e^−^ and h^+^ triggered by US, thus endowed Cu/TiO_2_ nanosonosensitizers with excellent SDT capability.^[^ ^22^ ^]^

The ROS types generated by single‐atom Cu/TiO_2_ nanosonosensitizers under US irradiation were further detected by the typical electron spin resonance (ESR) using 5‐tert‐butoxycarbonyl‐5‐pyrroline oxide (BMPO) and 2,6,6‐tetramethylpiperidine (TEMP) as the ROS trapping agents. As shown in Figure 2h, compared with other control groups, single‐atom Cu/TiO_2_ nanosonosensitizers generated a stronger characteristic signal of 1:2:2:1 (hydroxyl radicals) under US radiation and in the presence of H_2_O_2_ (100 µm). Similarly, the TiO_2_ occupied with Cu single atoms under ultrasonic radiation also produced a stronger characteristic signal of 1:1:1 (singlet oxygen radicals) than pure TiO_2_ (Figure 2i). The above results jointly explained that ultrasonic radiation promoted the efficiency of the Cu atom‐based Fenton‐like reaction, and the existence of the Cu single atoms simultaneously boosted the sonodynamic effect, which was consistent with the aforementioned results of TMB and DPBF absorption spectra.

As a powerful tool to explain the physical and chemical mechanism of biological materials, the density functional theory (DFT) calculations were conducted. To shed light on the internal mechanism of the enhanced reactive oxygen species production of Cu‐doped TiO_2_, the geometry optimizations, energy calculations, and electronic properties of the pure TiO_2_(001) facet and Cu‐doped TiO_2_(001) facet (Figure 2j) were calculated by using DFT calculations. It is anticipated that the Cu heteroatom contributes to the rearrangement of electron distribution on the surface, and the charge densities of the TiO_2_(001) facet before and after doping (Figure 2k) confirmed that Cu on the TiO_2_(001) surface attracted a number of electrons, which would facilitate the transfer of electrons and lead to a higher POD‐like catalytic activity. To prove the feasibility of H_2_O_2_ adsorption and decomposition processes on the TiO_2_(001)‐Cu facet, the catalytic reaction route and corresponding energy diagram were illustrated in Figure 2l,m. The H_2_O_2_ tended to adsorb on the facet with an energy drop of 0.48 eV, which was energy favorable and demonstrated the possibility of H_2_O_2_ adsorption. Then, a decomposition process of H_2_O_2_ proceeded with an energy drop of 0.20 eV. The H_2_O_2_ decomposed into OH* adsorbed on the surface by forming a Ti—O—Cu bond with bond lengths of 1.96 and 2.08 Å for the Ti—O and Cu—O bond respectively, and a ·OH to participate in the subsequent oxidation reaction in the organism. Under the acidic microenvironment of the tumor, the remaining OH* combined with the approaching H^+^ to produce an H_2_O adsorbed on the surface by forming a Ti—O bond of a bond length of 2.20 Å with an energy drop of 3.38 eV. Finally, the TiO_2_(001)‐Cu facet was recovered after the desorption and removal of H_2_O with low endothermic energy of 0.79 eV. These DFT results demonstrated that the aforementioned reaction route was reasonable and provided a feasible theory foundation for clarifying the proposed assistance role of Cu on the POD‐like catalytic activity of TiO_2_.

### 2.3 In Vitro Synergistic Sono/Chemo‐Dynamic Catalysis against TNBC Tumor Cells by Single‐Atom Cu/TiO_2_‐PEG Nanosonosensitizers

**Figure 3:**
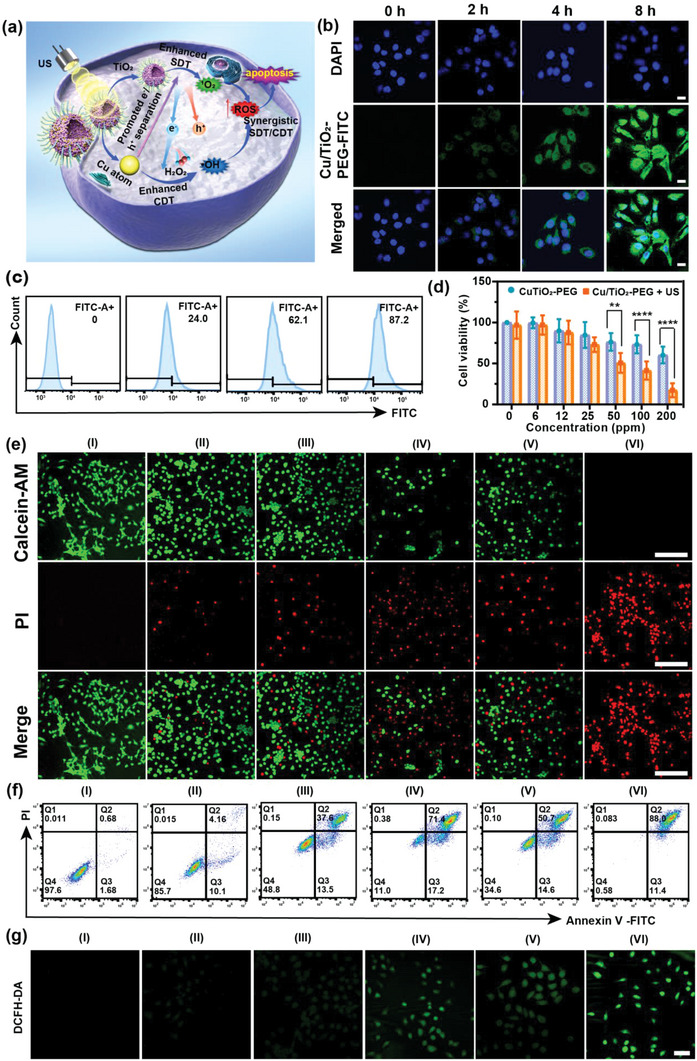
In vitro single‐atom Cu/TiO_2_‐PEG‐based synergistic therapies of TNBC. a) Schematic diagram of synergistically augmented sono/chemo‐dynamic therapy mediated by single‐atom Cu/TiO_2_‐PEG nanosonosensitizers. b) CLSM images of 4T1 cancer cells co‐incubated with FITC‐labeled single atom Cu/TiO_2_‐PEG nanosonosensitizers at varied time points (0, 2, 4, and 8 h) (Scale bars: 20 µm). c) Intracellular uptake of Cu/TiO_2_‐PEG after different intervals of co‐incubation (0, 2, 4, and 8 h) detected by flow cytometry analysis. d) Relative cell viabilities of 4T1 cancer cells after co‐incubation with different concentrations (0, 6, 12, 25, 50, 100, and 200 ppm) of single‐atom Cu/TiO_2_‐PEG nanoparticles for 24 h with or without US irradiation. The US irradiation parameters were set as 1.0 MHz, 1.0 W cm^−2^, 50% duty cycle, and 5 min. e) CLSM images of 4T1 cancer cells co‐incubated with Cu/TiO_2_‐PEG or TiO_2_‐PEG after varied treatments by stained with calcein‐AM (green fluorescence) and PI (red fluorescence) (Scale bars: 100 µm). f) Flow‐cytometry apoptosis assays of 4T1 cancer cells after different treatments followed by staining with Annexin V‐FITC and PI. g) CLSM observation of ROS generation in 4T1 cancer cells stained with DCFH‐DA. (Scale bars: 50 µm). (I: control, II: only US, III: TiO_2_‐PEG, IV: TiO_2_‐PEG + US, V: Cu/TiO_2_‐PEG, VI: Cu/TiO_2_‐PEG + US). *P< 0.05, **P< 0.01, ***P<0.001, ****P<0.0001 and ns for non‐significant.

The single‐atom Cu/TiO_2_‐PEG nanosonosensitizers produced a favorable cell‐killing effect on TNBC tumor cells due to the synergism of sonocatalysis and chemical catalysis (**Figure** **3**a). The nanoscale size of Cu/TiO_2_‐PEG nanoparticles ensured that they could be internalized into cancer cells. The intracellular uptake of FITC‐labeled Cu/TiO_2_‐PEG nanoparticles into 4T1 breast cells at different incubation durations (0, 2, 4, and 8 h) was detected by laser confocal microscopy (CLSM) (Figure 3b) and flow cytometry (Figure 3c; Figure S8, Supporting Information). After co‐incubation for 4 h, the green fluorescence of FITC‐labeled Cu/TiO_2_‐PEG could be directly observed in the cytoplasm of 4T1 cancer cells, where the blue fluorescence represented the DAPI‐stained nucleus. According to the flow cytometry results, the trend of average fluorescence intensity of FITC after different durations by flow cytometry was consistent with that observed by CLSM.

The effect of Cu/TiO_2_‐PEG nanosonosensitizers on the viability of 4T1 cells was calculated via the typical CCK‐8 assay. Cu/TiO_2_‐PEG nanosonosensitizers displayed dose‐dependent cytotoxicity to 4T1 cells. The inhibition rate of 4T1 cells under US irradiation by Cu/TiO_2_‐PEG reached nearly 82.65 ± 8.50% at the Ti concentration (200 ppm), which was significantly higher than that without US irradiation (39.67 ± 10.19%) (Figure 3d). To further visualize the live and dead cells, 4T1 cells were stained with calcein‐AM and propidium iodide (PI) solutions after different treatments for CLSM imaging. The CLSM results showed that slight cell damage was observed when receiving either SDT alone or CDT alone, whereas the synergistic treatment triggered the greatest degree of cell death (Figure 3e), which was also determined by flow cytometry based on the typical Annexin V‐FITC and PI co‐staining (Figure 3f; Figure S9, Supporting Information). As shown in Figure 3f and Figure S9 (Supporting Information), the percentage of apoptotic cells in the Cu/TiO_2_‐PEG + US group was 99.4% (early stages apoptosis 11.4%, late stages apoptosis 88.0%), much higher than TiO_2_‐PEG + US group (86%) and other groups, which further confirmed that significant cancer‐cell death in the form of apoptosis can be induced via synergistic CDT and SDT effects by Cu/TiO_2_‐PEG under US irradiation. 2′,7′‐dichlorofluorescin diacetate (DCFH‐DA) could serve as a ROS fluorescence probe to verify the ROS generation induced by US activation in the presence of Cu/TiO_2_‐PEG nanosonosensitizers. Strong green fluorescence was observed in 4T1 cancer cells treated with Cu/TiO_2_‐PEG + US, which was much brighter than that of the TiO_2_‐PEG + US group. The mean fluorescence intensity of Cu/TiO_2_‐PEG + US was much higher than other groups, indicating that abundant ROS was produced during the sono/chemical catalytic process assisted by single atom Cu/TiO_2_ nanosonosensitizers (Figure 3g; Figure S10, Supporting Information). In addition, the production of ROS was further confirmed by flow cytometry (Figure S11, Supporting Information), and the trend of fluorescence intensity was consistent with that observed by CLSM.

### 2.4 Gene Sequencing Analysis

**Figure 4:**
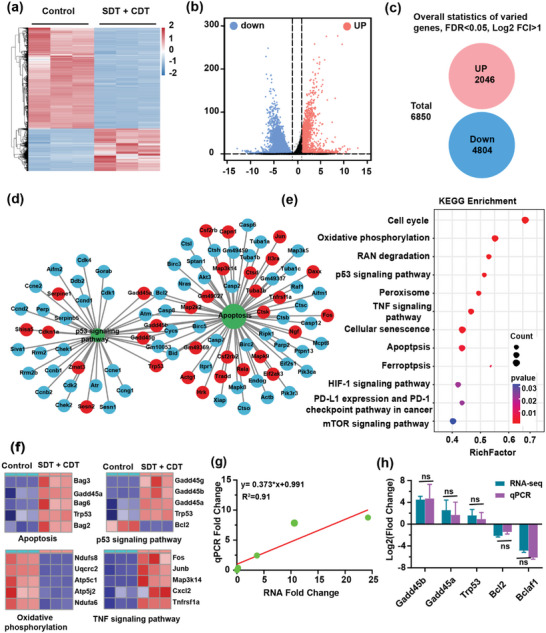
The underlying mechanism of enhanced sono/chemo‐dynamic treatments of breast cancer with single‐atom Cu/TiO_2_‐PEG nanosonosensitizers by transcriptome high‐throughput sequencing. a) Heat map and b) volcano map of expressed genes involved in the synergistically therapeutic progress. c) Overall statistics of 6850 varied genes including 2046 up‐regulations and 4804 down‐regulations. d) Functional protein interaction network analysis of differentially expressed genes. e) Bubble diagram of differential genes expression in KEGG analysis. f) Heatmaps of differentially expressed genes associated with P53, apoptosis, oxidative phosphorylation, and TNF signaling pathway. g) Correlation analysis between RNA‐seq and qRT‐PCR genes differential expression. h) Comparative analysis of RNA‐seq and qRT‐PCR gene expression (*n* = 3). *P< 0.05, **P< 0.01, ***P<0.001, ****P<0.0001 and ns for non‐significant.

To further explore the underlying mechanism regarding the synergistic SDT and CDT effects of single‐atom Cu/TiO_2_‐PEG nanosonosensitizers, we analyzed the variations of mRNAs levels of 4T1 cancer cells with or without synergistic therapy by high‐throughput sequencing. Briefly, 4T1 cells treated without any treatment and treated with Cu/TiO_2_‐PEG nanosonosensitizers under US irradiation were labeled as control and SDT + CDT group, respectively. The heat map and principal component analysis (PCA) showed that there were distinct transcriptomic diversities between the control and SDT + CDT groups (**Figure** **4**a; Figure S12, Supporting Information). More than 6850 genes were widely regulated in the SDT + CDT group, compared to the control group (*P* < 0.05), including 2046 up‐regulated genes and 4804 down‐regulated genes (Figure 4b,c). Among them, synergistic treatment induced the up‐regulation of p53 and its downstream genes, such as Trp53, gaddb45a, and gaddb45b, and the down‐regulation of Bcl‐2 family genes, such as Bcl‐2, which indicated that the above genes might participate in the synergistically therapeutic effects of single‐atom Cu/TiO_2_‐PEG nanosonosensitizers against cancer cells. The network analysis of the above‐mentioned genes further illustrated the interaction of key genes (Figure 4d). The data also revealed that the up‐regulation of Trp53, Gadd45a, Gadd45b, and the down‐regulation of Bcl‐2 played an important role in promoting apoptosis of 4T1 tumor cells.

For better elucidating the biological functions of the synergistic SDT and CDT effects of single‐atom Cu/TiO_2_‐PEG nanosonosensitizers, the mRNAs with significantly altered expression levels were analyzed by Gene Ontology (GO), including biological process (BP), molecular function (MF) and cellular component (CC) (Figure S13, Supporting Information). Relevant signaling pathways include the cellular response to stress, DNA damage stimulus, positive regulation of protein dephosphorylation signaling pathway, *etc*. Based on the above results, the enrichment analysis was performed on the Kyoto Encyclopedia of Genes and Genomes (KEGG) signaling pathway (Figure 4e). The expression of genes related to the p53 signaling pathway and apoptosis pathway was noticeably altered in the SDT + CDT group. Single‐atom Cu/TiO_2_‐PEG generated more ROS and caused extensive DNA damage, which activated the p53 signaling pathway and promoted apoptosis. Moreover, under oxidative stress, pro‐apoptotic gene p53 could further promote apoptosis by controlling the metabolism of cancer cells.^[^ ^23^ ^]^ After synergistic treatment, the expression of p53 on 4T1 cancer cells was prominently up‐regulated, indicating that synergistic treatment could activate the expression of p53 and its downstream genes and accelerate cell apoptosis (Figure 4f). From Figure 4f, in addition to the significant differences in the expression of p53 and apoptosis‐related genes, oxidative phosphorylation (Ndufs8, Ndufa6, Uqcrc2, Atp5c1, Atp5j2, etc.) and tumor necrosis factor (Fos, Junb, Map3k14, Cxcl2, Tnfrsf1a, etc.) are also altered, indicating that Cu/TiO_2_‐PEG‐mediated synergistic SDT and CDT could play a role in the above genes. Cu/TiO_2_‐PEG nanosonosensitizers with the assistance of US could efficiently promote the production of ROS during synergistic therapy, and then inhibit oxidative phosphorylation in a ROS‐dependent manner, thereby inducing energy crisis and apoptosis of cancer cells. At the same time, Cu/TiO_2_‐PEG nanosonosensitizers could increase the permeability of mitochondria in cells by the action of tumor necrosis factor (TNF signaling pathway), and promote the generation of ROS, thus leading to apoptosis of cancer cells.

In order to verify the genes expression difference of RNA‐seq, we selected 5 genes with a differential gradient in expression difference for quantitative reverse transcription polymerase chain reaction (qRT‐PCR) verification (*n* = 3), including three up‐regulated genes (Trp53, Gadd45a, Gadd45b) and two down‐regulated genes (Bcl‐2, Bclaf1). The results were consistent with the gene expression of RNA‐seq, indicating that the gene differential expression analysis of RNA‐seq was highly reliable (Figure 4g,h).

### 2.5 In Vivo Synergistic Single‐Atom Fenton Catalysis and Sonodynamic Therapy Against TNBC by Cu/TiO_2_‐PEG

**Figure 5:**
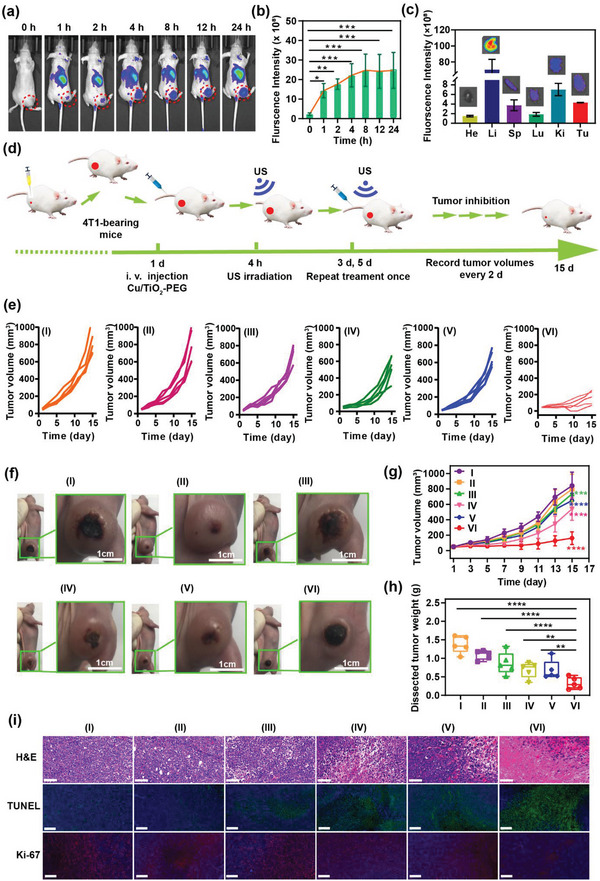
Single‐atom Cu/TiO_2_‐PEG nanosonosensitizers for mutually enhanced sono/chemo‐dynamic therapy of tumor in vivo. a) In vivo fluorescence (FL) imaging of the subcutaneous 4T1 tumor models, after injection of Cu/TiO_2_‐PEG via tail vein at different time points (0, 1, 2, 4, 8, 12, and 24 h). b) The changes in FL signal intensities of the tumors at different time points (*n* = 3). c) Ex vivo FL images and intensities of main organs and tumors form mice 24 h post‐injection. d) Schematic diagram of the establishment of 4T1 tumor‐bearing mice and evaluation of the therapeutic effect of Cu/TiO_2_‐PEG under US irradiation. e) Individual tumor growth curve of mice after different treatments. f) Digital images of tumors from each group on the 15^th^ day. g) Time‐dependent tumor‐volume curves in all groups. h) Excised tumor weight of each group. i) H&E staining (scale bar: 100 µm), TUNEL staining and Antigen Ki‐67 staining (scale bar: 200 µm) in tumors of each group after various treatments. (I: control, II: only US, III: TiO_2_‐PEG, IV: TiO_2_‐PEG + US, V: Cu/TiO_2_‐PEG, VI: Cu/TiO_2_‐PEG + US). *P< 0.05, **P< 0.01, ***P<0.001, ****P<0.0001 and ns for non‐significant.

The xenograft model of the 4T1 breast tumor was established in nude mice to assess the tumor accumulation and biodistribution in vivo. The Cys 5.5‐labeled Cu/TiO_2_‐PEG (Cys 5.5‐Cu/TiO_2_‐PEG) nanoparticles were used as a contrast agent for the fluorescence imaging system (IVFIS) to investigate their biodistribution through an in vivo IVFIS. The FL imaging was performed on 4T1 tumor‐bearing mice at different time points after intravenous injection of Cys 5.5‐Cu/TiO_2_‐PEG nanoparticles (**Figure** **5**a). As shown in Figure 5b, the FL signal intensity within the tumor region gradually increased from 1 h to 24 h after the injection of nanosonosensitizers, and reached a peak at 24 h (24.7 ± 9.18 × 10^9^). In 24 h post‐injection, tumor nodules and major organs were collected for ex vitro FL imaging (Figure 5c). It is not surprising that large quantities of nanoparticles accumulated within the liver due to the strong uptake capacity of the reticuloendothelial system. The Cu/TiO_2_‐PEG accumulated significantly in tumor sites, which is based on the typical enhanced permeability and retention (EPR) effects. To demonstrate the efficient accumulation of Cu/TiO_2_‐PEG in the tumor tissues of 4T1 tumor‐bearing mice, the tumor tissues were harvested for inductively coupled plasma optical emission spectrometer (ICP‐OES) analysis at 4, 24, and 48 h after intravenous injection. As displayed in Figure S14 (Supporting Information), the Cu/TiO_2_‐PEG distributed into the tumor region reached nearly 2.84% ± 0.83%ID/g at 48 h post‐injection because of the typical enhanced permeability and retention effect, which was still close to the content in the tumor at 4 h post‐injection (3.18 ± 0.37% ID/g).

The in vivo biosafety and biocompatibility of Cu/TiO_2_‐PEG nanosonosensitizers were systematically assessed to ensure their clinical translation. Twenty female ICR mice were randomly divided into 4 groups and then injected with different doses of Cu/TiO_2_‐PEG (doses: 0, 1.25, 2.5, and 5 mg kg^−1^) via tail vein. The mice were sacrificed after 30 days, and blood samples and major organs were collected for blood biochemical and histological identification (H&E) analysis, respectively. Compared with the control group, a series of blood indexes of the Cu/TiO_2_‐PEG treatment groups were not significantly different from those without any treatment (Figure S15, Supporting Information). No obvious signs of organ damage and inflammatory lesions were found in the H&E staining analysis (Figure S16, Supporting Information). All the results suggested the high biocompatibility of the single‐atom Cu/TiO_2_‐PEG nanosonosensitizers, which provides a basis for safe treatment.

Inspired by the in vitro satisfactory effects, the in vivo synergistic single‐atom Fenton catalysis and sonodynamic therapy were systematically evaluated by injecting single‐atom Cu/TiO_2_‐PEG nanosonosensitizers into 4T1 breast tumor‐bearing mice via tail vein. Thirty female tumor‐bearing mice were randomly divided into 6 groups (*n* = 5 mice in each group), including (I) control group (saline), (II) US group, (III) TiO_2_‐PEG group, (IV) TiO_2_‐PEG + US group, (V) Cu/TiO_2_‐PEG group, (VI) Cu/TiO_2_‐PEG + US group. After 4 h of intravenous injection of the corresponding nanoparticles at the dose of 5 mg kg^−1^, these mice were treated according to the treatment protocol as illustrated in Figure 5d. The body weight and tumor volume were noted every two days. The treatment was repeated once under the same conditions on the third and the fifth day. No obvious changes in the body weight of all mice were monitored during the observation process (Figure S17, Supporting Information). Compared with the TiO_2_‐PEG group, the growth of tumors in the Cu/TiO_2_‐PEG group was slightly inhibited, and the tumor inhibition rates of the TiO_2_‐PEG group and Cu/TiO_2_‐PEG group were 2.70% and 11.31%, respectively. Comparatively, the Cu/TiO_2_‐PEG + US group achieved the highest inhibition rate of 70.35%, which was significantly higher than that of the TiO_2_‐PEG + US group (30.91%) (Figure 5e,f; Figure S18, Supporting Information). The curves of tumor volume further solidly demonstrated that the combination of SDT and single‐atom catalysis achieved a satisfactory synergistic efficiency to inhibit TNBC tumor growth (Figure 5g). Besides, by comparing the weight of tumors dissected after treatment as shown in Figure 5h, it was found that mice treated with Cu/TiO_2_‐PEG + US obtained more significant tumor inhibition than the other groups of mice.

To further confirm the anti‐tumor mechanism of synergistic single‐atom Fenton catalysis and SDT, tumor sections of mice in each group were collected at the end of the treatments for the hematoxylin and eosin staining (H&E), terminal deoxynucleotidyl transferase dUTP nick end labeling (TUNEL) and Ki‐67 antibody staining (Figure 5i). As shown in the tumor sections stained with H&E, the changes in tumor cells morphology or state in the control group, only US group and TiO_2_‐PEG group were negligible, whereas the Cu/TiO_2_‐PEG + US group showed obvious changes in tumor cell morphology, including nuclear pyknosis, nuclear rupture, and nuclear lysis, which indicated the substantial necrosis of cancer cells. The TUNEL staining was carried out to detect the apoptosis of tumor cells. It was found that tumors in the Cu/TiO_2_‐PEG + US group had significantly elevated apoptosis levels, which were much higher than that of other groups. The Ki‐67 antibody staining was commonly conducted to evaluate the proliferation of cancer cells, which also proved that the proliferation index of the Cu/TiO_2_‐PEG + US group declined more noticeably than the other groups. The above staining of tumor tissue sections confirmed the efficient anti‐tumor effect of Cu/TiO_2_‐PEG as the single‐atom catalyst and sonosensitizer for synergistic sono/chemo‐dynamic therapy. The main organs (heart, liver, spleen, lung, and kidney) were further stained with H&E to evaluate the therapeutic biosafety after different treatments. No obvious histopathological changes were found in the control group and other therapeutic groups, indicating the high therapeutic biosafety of this synergistic modality (Figure S19, Supporting Information).

## 3 Conclusions

In summary, we proposed a dual synergistic sono/chemo‐catalytic strategy by rationally constructing a single‐atom Cu‐based hollow TiO_2_ nanosonosensitizer for the non‐invasive and efficient suppression of breast tumors. The Cu single‐atoms occupied the most stable Ti vacancies in the hollow TiO_2_ nanosonosensitizers, which enabled considerable catalytic ROS generation and the subsequent remarkable antitumor performances in response to specific US irradiation and tumor microenvironment. Simultaneously, the presence of Cu single‐atoms could significantly improve the separation efficiency of electrons (e^−^) and holes (h^+^) triggered by US and then accelerated the ROS generation. In the in vivo attempts, a comprehensive tumor suppression rate of 70.35% has been achieved under the synergistically augmented sono/chemo‐catalytic nanotherapeutics using the single‐atom Cu/TiO_2_‐PEG nanosonosensitizers. The successful evidence of efficient sono/chemo‐nanocatalyst in refractory tumor suppression can provide a representative paradigm for non‐invasive nanocatalytic medicine by initiating different types of tumor‐therapeutic reactions, such as chemocatalysis and sonocatalysis.

## Conflict of Interest

The authors declare no conflict of interest.

## Supporting information

Supporting Information

## Data Availability

The data that support the findings of this study are available from the corresponding author upon reasonable request.
