# Supplementary material for: Single Atom‐Doped Nanosonosensitizers for Mutually Optimized Sono/Chemo‐Nanodynamic Therapy of Triple Negative Breast Cancer
Source: Adv Sci (Weinh). 2023 Jan 16;10(6):2206244. doi: 10.1002/advs.202206244 (PMC9951334; doi:10.1002/advs.202206244)
Supplement: Supplementary file 1 — Supporting Information [file ADVS-10-2206244-s001.pdf]

Supporting Information

Single Atom-Doped Nanosonosensitizers for Mutually Optimized Sono/Chemo-Nanodynamic Therapy of Triple Negative Breast Cancer

Qiqing Chen, Min Zhang, Hui Huang, Caihong Dong, Xinyue Dai, Guiying Feng, Ling Lin, Dandan Sun, Dayan Yang, Lin Xie, Yu Chen, Jia Guo,* and Xiangxiang Jing**

Q. Chen, M. Zhang, G. Feng, L. Lin, D. Sun, D. Yang, L. Xie, Prof. X. Jing

Department of Ultrasonography, Hainan General Hospital/Hainan Affiliated Hospital of Hainan Medical University, Haikou, 570311, P. R. China. E-mail: ljjxx@hainmc.edu.cn (X. Jing).

Prof. J. Guo

Shuguang Hospital Affiliated to Shanghai University of Traditional Chinese Medicine, Shanghai, 201203, P. R. China. Email: sg_jia_guo@shutcm.edu.cn (J. Guo).

Dr. X. Dai, H. Huang, Prof. Y. Chen

Materdicine Lab, School of Life Sciences, Shanghai University, Shanghai, 200444, P. R. China. Email: chenyuedu@shu.edu.cn (Y. Chen).

Dr. C. Dong

Department of Ultrasound, Zhongshan Hospital, Fudan University, and Shanghai Institute of Medical Imaging, Shanghai, 200032, P. R. China.

A: Experimental Section

Materials: Titanium (IV) *n*-butoxide (TBOT) was purchased from Strem Chemicals, copper (II) chloride dihydrate ($CuCl_2{\cdot}2H_2O$), tetraethyl orthosilicate (TEOS), polyvinylpyrrolidone (PVP) (*M*w = 55,000), sodium hydroxide (NaOH), ammonia solution (28 - 30 wt%), acetonitrile, anhydrous ethyl alcohol were purchased from Sinopharm Chemical Reagent Co., Ltd. Methoxypolyethylene glycol amine 2000 (NH_2-PEG_{2000}) was purchased from Ponsure Biological Co., Ltd. (Shanghai, China) 1,3-diphenylisobenzofuran (DPBF) and 3,3', 5,5'-tetramethylbenzidine (TMB) were obtained from Ruicheng BioTech (Shanghai, China). The Cell Counting Kit-8 (CCK8 assay kit), Calcein AM and propidium iodide (PI), Annexin V-FITC Apoptosis, 2’,7’-Dichlorofluorescin diacetate (DCFH-DA), 5-tert-butoxycarbonyl-5-pyrroline oxide (BMPO) and 2,6,6-tetramethylpiperidine (TEMP) were purchased from Dojindo Chemical Technologies (Shanghai, China). Dulbecco's modified eagle medium (DMEM), fetal bovine serum (FBS), trypsin solution, and streptomycin/penicillin (1%) were purchased from Procell Biotechnology Co., Ltd. (Wuhan, China). All chemical reagents were used without further depuration.

Characterization: TEM, HRTEM, and HAADF-STEM were adopted for morphology and elemental distribution analysis on a JEOL ARM-300F electron microscope operated at 300 kV. X-ray diffraction (XRD) was conducted on Rigaku D/MAX-2200 PC-ray power diffractometer with Cu Kα radiation (40 mA, 40 kV, and λ = 1.54 Å) scanning with a 2θ ranges from 10-90°. DLS was conducted on Malvern Zetasizer Nanoseries (Nano ZS90) for hydrodynamic particle size determination. XPS spectrum was obtained by a Thermo Fisher Scientific ESCALAB 250Xi X-ray spectrometer (Thermo Fisher Science, Waltham, MA, USA). The X-ray tube with AlKα-radiation (1486.6 eV) was used as the ionizing radiation source. UV-*vis* absorption spectra were measured by using a SynergyHTX microplate reader

(BioTek Instruments, United States). ESR experiment was conducted on a JEOL-FA200 spectrometer. Confocal laser scanning microscopy images (CLSM) were acquired by FV1000 Olympus Co. US irradiation for sonodynamic anti-tumor therapy was performed by an Intelligent Transport Ultrasound (Chattanooga Group, USA).

Synthesis and Surface NH_2-mPEG$_{2000}$ Modification of Cu/TiO_2 Nanoparticles (Cu/TiO_2-PEG): The single-atom Cu/TiO_2 nanoparticles were synthesized according to the published literature. [1] Firstly, spherical silica (SiO_2) nanoparticles were synthesized under alkaline conditions. The TEOS (0.86 mL) was dissolved in a mixed solution, which contained H_2O (4.3 mL), ammonia (0.6 mL), and ethanol (23 mL). Then, the mixed solution was stirred at room temperature for 6 hours. The SiO_2 nanoparticles were collected by centrifugation and dispersing in ethanol (40 mL). Secondly, ammonia (0.4 mL, 28 - 30 wt%) and pure acetonitrile (14 mL) were mixed with the above SiO_2 nanoparticles in solution and stirred for 30 min (called solution a). Then, TBOT (0.8 mL) was added to acetonitrile (2 mL) and 6 mL absolute ethanol solution (called solution b). Solution b was slowly dripped to solution a and kept stirring for 3 h to cover with TiO_2. The products were centrifuged and washed with H_2O several times to obtain SiO_2@TiO_2 nanoparticles and dispersed in H_2O (40 mL). Thirdly, $CuCl_2 \cdot 2H_2O$ (1 mL, 6 mg/mL) solution was added to the previously SiO_2@TiO_2 nanoparticles and stirred uniformly for several hours. After stirring, the SiO_2@CuO_x/TiO_2 nanoparticles were obtained by centrifugation and washing with H_2O and dispersed in H_2O (40 mL). Fourthly, PVP (400 mg) was dispersed in H_2O (20 mL) to obtain an aqueous PVP solution, which was added to SiO_2@CuO_x/TiO_2 solution. After the addition of PVP, the mixture was stirred for 8 h to make the adsorption of PVP onto SiO_2@CuO_x/TiO_2 nanoparticles. After the adsorption of PVP, the products were separated by centrifugation and scattered in H_2O (8.6 mL) and ethanol (46 mL) solution. Then TEOS (1.6 mL) and ammonia (1.2 mL, 28 - 30 wt%)

were added to the above solution, then formed SiO_2 coating (SiO_2@CuO_x/TiO_2@SiO_2 nanoparticles). After 4 h, the products were centrifuged and dried overnight in a low-temperature dryer and grind in mortar for uniformity. Fifthly, to spatially redistribute Cu single-atoms, the dried nanoparticles were calcined at 900 °C for 2 h to yield SiO_2@Cu/TiO_2@SiO_2 nanoparticles. Finally, the nanoparticles were dispersed in NaOH solution (20 mg mL^{-1}) and heated to 90 ℃ by uniformly stirring. After 6 h, the products were centrifuged and washed with H_2O to yield an aqueous Cu/TiO_2 solution. The products were frozen overnight at a low temperature and used for characterization further use.

For enhancing the stability and biosafety of Cu/TiO_2 nanoparticles under physiological conditions, biocompatible methoxypolyethylene glycol amine 2000 (NH_2-$mPEG_{2000}$) was grafted onto the surface of Cu/TiO_2 nanoparticles. 5 mL of Cu/TiO_2 nanoparticles solution ([Ti]: 400 ppm) and NH_2-$mPEG_{2000}$ (40 mg) were added into a necked bottle, which was sonicated for 60 min and then stirred overnight. The Cu/TiO_2-PEG was collected by centrifugation and washed several times with H_2O to remove the non-attached PEG.

In Vitro ROS Generation of Cu/TiO_2 by US Activation: 3, 3', 5, 5'-tetramethylbenzidine (TMB) was used to verify the occurrence of the Fenton reaction, based on the production of ·OH. Cu/TiO_2 (100 μL, 300 ppm) was mixed with TMB in acidic solution (0.01 mM TMB testing solution 100 μL, 100 μM H_2O_2 solution 100 mL, PBS buffer 1.7 mL, pH 6.5). The absorbance change of the TMB working solution at 652 nm was recorded every minute, for a total of 20 minutes. Similar operations were performed for the other groups (control and TiO_2). Meanwhile, the absorbance changes of the TMB working solution with or without US irradiation (power density: 1.0 W cm^{-2}, duty cycle: 50 %, 5 min) were recorded after 20 minutes under different concentrations of Cu/TiO_2 (0, 50, 100, 150, 200, 250, and 300 ppm).

A singlet oxygen probe, 1,3-diphenylisobenzofuran (DPBF), was employed for characterizing the effects of US-activated Cu/TiO_2 nanosonosensitizers on ROS-generating efficacy *in vitro*. In a typical assay, DPBF (2 mg mL^{-1}, 40 μL) and Cu/TiO_2 nanoparticles (100 μL, Ti concentration: 200 ppm) were mixed with DMF (2.86 mL). Then, the mixed solution was subjected to irradiation by US (50 % duty cycle, 1 MHz, 1.0 w cm^{-2}) for various lengths of time in the dark (0, 2.5, 5, 7.5, 10, and 12.5 min). The variation in absorbance of DPBF at 416 nm was identified using the UV-*vis*-NIR spectrum. Meanwhile, the ROS generation of different concentrations of Cu/TiO_2 was verified under US activation (0, 50, 100, 150, 200, and 250 ppm).

Electron Spin Resonance (ESR) Spectra Test in Vitro: Utilizing electron spin resonance (ESR), Cu/TiO_2 nanosonosensitizers during US activation produced different forms of ROS. The activity of •OH was investigated by ESR using a spin-trapping agent precisely (BMPO). For purpose of measuring the ESR, solutions containing TiO_2 or Cu/TiO_2 (Ti concentration: 200 ppm), 100 mM H_2O_2, and 250 mM BMPO were combined. The solutions were then subjected to US irradiation (1.0 W cm^{-2}, 50% duty cycle, 1.0 MHz, 5 min). Additionally, the production of $^1O_2$ was confirmed using the TEMP (100 mM). TiO_2 or Cu/TiO_2 (Ti concentration: 200 ppm) and TEMP (250 mM) were combined, and were subjected to US radiation (1.0 W cm^{-2}, 50% duty cycle, 1.0 MHz) for 5 min. The $^1O_2$ signal was picked up by the ESR spectrometer.

Computational Details: The atomic structure optimizations and electronic structure calculations of TiO_2 (001) facets were performed based on the DFT calculations within the Perdew-Burke-Ernzerhof (PBE) functional [2] by using the Vienna Ab initio Simulation Package (VASP). [3] Taking into account the influence of Van der Waals interactions, DFT-D3 function was employed. [4] The electronic plane wave interception energy was set to 350

eV, and these configurations were optimized until the energy differences were converged within 10^{-4} eV and the forces of all atoms were less than 0.01 eV/Å. The vacuum layers between neighboring images were set to be larger than 15 Å, which was enough to avoid the interactions between neighboring images. The models were constructed of 2×2×5 TiO_2 unit cells with periodic boundary conditions (PBC) in plane and the bottom two layers were fixed to simulate the semi-infinite substrate bulk. The 5×5×1 k-point Gamma meshes were performed for these models.

Cell Culture: The Cell Bank of the Chinese Academy of Sciences in Shanghai provided the 4T1 mouse breast cancer cell line. The 4T1 Cells were grown in DMEM with 10% foetal bovine serum (FBS), 1% penicillin/streptomycin, and 5% CO_2 humidified conditions at 37 °C.

In Vitro Cytotoxicity Assay: For the *in vitro* cytotoxicity tests, 4T1 cells were employed. 96-well plates were selected for culturing 4T1 cells at 1×10^4 cells per well, in DMEM supplemented with 10% FBS, 1% penicillin/streptomycin, and a humidifier controlled at 5% CO_2 and 37 °C. To evaluate the cytotoxicity of synergistic single-atom catalysis and SDT using the CCK-8 assays, 4T1 cells were cultured with 100 μL of DMEM containing different concentrations of Cu/TiO_2-PEG ([Ti]: 0, 6, 12, 25, 50, 100 and 200 ppm) in 96 well plates and were irradiated with the US after 4 h of co-incubation. Then, cell viabilities were then subjected to a standard CCK-8 assay after 24 h. After the solution was washed by PBS thrice, 100 μL of DMEM containing 10 μL of CCK8 solution was added to each well, and co-incubated for 1 h, which was measured in a SynergyHTX microplate reader at 450 nm. US irradiation conditions were adjusted as 50 % duty cycle, 1.0 MHz, and 1.0 W cm^{-2}, 5 min.

Endocytosis Analysis of Cu/TiO$_2$-PEG as Observed via Flow Cytometry and CLSM: To track the endocytosis of Cu/TiO$_2$-PEG, CLSM was employed. Seeding of 1× 10^5 4T1 cells was made into dishes made specifically for CLSM, and the dishes were then cultured in a humid incubator. Then, 1 ml of DMEM containing FITC-labeled Cu/TiO$_2$-PEG nanosonosensitizers was added in place of the original media. After different incubation durations (0, 2, 4, and 8 h), CLSM was carried out after staining with DAPI for 15 min and washing with PBS.

To further perform the endocytosis of Cu/TiO$_2$-PEG nanosonosensitizers by the flow cytometry, 6-well plates were used to plate 4T1 cells at 3 × 10^5 cells per well and 2 ml of DMEM supplemented with FITC-labeled Cu/TiO$_2$-PEG nanosonosensitizers was used to replace the medium for various incubation periods (0, 2, 4 and 8 h). Specific test tubes were used to collect 4T1 cells, and the average fluorescence intensities of FITC-labeled Cu/TiO$_2$-PEG in 4T1 cells were analyzed by flow cytometry.

In Vitro Synergistic Single-atom Catalysis and SDT Effects as Observed by Flow Cytometry and CLSM: In a dish designed for CLSM, 4T1 cancer cells were plated and set to co-incubate with the appropriate Cu/TiO$_2$-PEG nanosonosensitizers. The cells underwent a 24-hour treatment period using a variety of procedures, including the control group, the only US group, the TiO$_2$-PEG group, the TiO$_2$-PEG + US group, the Cu/TiO$_2$-PEG group, and the Cu/TiO$_2$-PEG + US group. For 5 min, the cells were treated with US (50% duty cycle, 1 MHz, and 1.0 W cm^{-2}). The media was then taken out and stained for 15 minutes with calcein-AM and PI solution. The monitoring of the cells was completed by CLSM, in which the dead cells were labeled with red and the living cells with green.

4T1 cells were cultured in a 6-well plate and grew to a percentage of 70 to 80% in order to analyze apoptosis using flow cytometry. As previously noted, cells were subjected to treatments after being cultivated for 24 hours. With 500 μL of binding buffer, cells were

collected and disseminated once more. FITC (15 μL) and Annexin V (5 μL) were utilized for labeling the living and dead cells for a duration of 20 min. Flow cytometry was ultimately used to identify cell apoptosis.

In Vitro Generation of ROS as Performed by CLSM and Flow Cytometry: In a CLSM-specific culture dish, 4T1 cells were seeded (1×10^5 cells per dish) and then distributed into six groups: control group, only US group, TiO_2-PEG group, TiO_2-PEG + US group, Cu/TiO_2-PEG, and Cu/TiO_2-PEG + US group. The Cu/TiO_2-PEG nanosonosensitizers were allowed to incubate with cells for a period of 4 h. Cells were subsequently treated with US (50 % duty cycle, 1 MHz, and 1.0 W cm^{-2}) for 5 min. Appropriate amounts of DCFH-DA were added to individual culture dishes for 20 min. Finally, CLSM was used for observing the cells.

The generation of ROS in 4T1 cells was detected using flow cytometry. Typically, 6-well culture plates were used to culture 4T1 cells (3×10^5 cells per well). The cells, which were treated as mentioned above, were collected and the fluorescence intensity of DCFH-DA was detected following centrifugation.

Analysis of Gene Sequencing: TRIzol (Beyotime Biotechnology, Nanjing) was taken to extract total RNA from 4T1 cells in the control group (n=3) and Cu/TiO_2-PEG+US group (n=3), following the instructions furnished by the manufacturer. The groups were labeled as the control group and SDT + CDT group, respectively. For RNA-seq analysis, the samples were subsequently dispatched to Gene Denovo Biotechnology Co., Ltd (Guangzhou, China). Illumina hiseq X10 (Illumina, San Diego, CA) was utilized for the construction of the library and subsequent sequencing. Gene Ontology (GO) terms and Kyoto Encyclopedia of Genomes (KEGG) signaling pathway analysis was carried out after enriching the differentially expressed genes.

In Vivo Fluorescence (FL) Imaging Performance of Cu/TiO_2-PEG: All experiments involving animals were proceeded following approval and strict adherence to the guidelines and regulations of the Animal Care Ethics Commission of Shanghai University. A fluorescence imaging system (IVFIS) was performed on the Xenogeny IVFIS Spectrum imaging system (Perkin Elmer, USA). For the FL imaging *in vivo*, the subcutaneous tumor models of 4T1 cells (1×10^6 cell/site) were established in female BALB/C nude mice. Following the growth of the tumor to a mean volume of approximately 70 mm^3, Mice were intravenously injected with Cys 5.5-labeled Cu/TiO_2-PEG nanosonosensitizers (2.5 mg kg^{-1}, 150 μL) *via* tail vein. The images were taken at 0, 1, 2, 4, 8, 12, and 24 h following injection. Subsequently, mice were euthanized and tumor nodules and key organs (heart, lung, kidney, spleen, and liver) were gathered for analysis of *ex vivo* FL intensities.

In Vivo Biocompatibility Evaluation: Random division of twenty female ICR mice resulted in four groups and assessed for the in vivo biocompatibility and biosafety studies (n = 5 per group), including (1) control (intravenous injection with 100 μL of saline), (2) 1.25 mg kg^{-1} (intravenous injection with 100 μL of Cu/TiO_2-PEG at a dose of 1.25 mg kg^{-1}), (3) 2.5 mg kg^{-1} (intravenous injection with 100 μL of Cu/TiO_2-PEG at a dose of 2.5 mg kg^{-1}), and (4) 5 mg kg^{-1} (intravenous injection with 100 μL of Cu/TiO_2-PEG at a dose of 5 mg kg^{-1}). After feeding for 30 days, twenty mice were euthanized, and blood specimens and main organs (heart, lung, kidney, spleen, and liver) were utilized for blood investigations and histopathological analysis, respectively.

In Vivo Synergistic Single-atom Catalysis and SDT of Cu/TiO_2-PEG under US Irradiation: For *in vivo* synergistic single-atom catalysis and SDT therapy, 4T1 cells suspended in 100 μL PBS were injected subcutaneously into the right back (1×10^6 cells/site) of the female

BALB/C nude mice. Random division of the mice was made into six groups following the growth of the tumor to a mean volume of about 70 mm^3 (5 mice in each group): (I) control group (treated with saline only), (II) only US group (only exposure to US irradiation for 5 min), (III) TiO_2-PEG group (TiO_2-PEG injected intravenously), (IV) TiO_2-PEG + US group (TiO_2-PEG injected intravenously followed by exposure to US irradiation for 5 min), (V) Cu/TiO_2-PEG group (Cu/TiO_2-PEG injected intravenously) and (VI) Cu/TiO_2-PEG + US group (Cu/TiO_2-PEG injected intravenously followed by exposure to US irradiation for 5 min). The US exposure conditions employed were 50% duty cycle, 1 MHz, and 1.0 W cm^{-2}. These mice were treated according to the protocol following intravenous injection of the corresponding nanoparticles at a dose equivalent to 5 mg kg^{-1}. The US exposure conditions employed were 50 % duty cycle, 1.0 MHz, and 1.0 W cm^{-2}. The body weight and tumor volume were carefully monitored every two days for 15 days. The treatment was repeated once under identical circumstances on the third and fifth days. Meanwhile, digital photographs of the tumor sites were taken. After treatment, a vernier caliper was employed for estimating the width (W) and length (L) of tumors every two days. The mathematical expression for computing the tumor volumes is given below: tumor volume (V) = $L \times W^2/2$. TUNEL, H&E, and Ki-67 antibodies were used to stain tumor tissues for histopathological analysis at the conclusion of treatment.

Statistical Analysis: All data are presented as mean ± standard deviation (s.d.). Statistical analysis was carried out by making use of GraphPad Prism software 8.0 (GraphPad Software). Two-way ANOVA and Student's two-tailed t-test were employed for determining the statistical significance of the comparison among the two groups (*$P < 0.05$, **$P < 0.01$, ***$P < 0.001$, ****$P < 0.0001$).

B: Supplementary figures

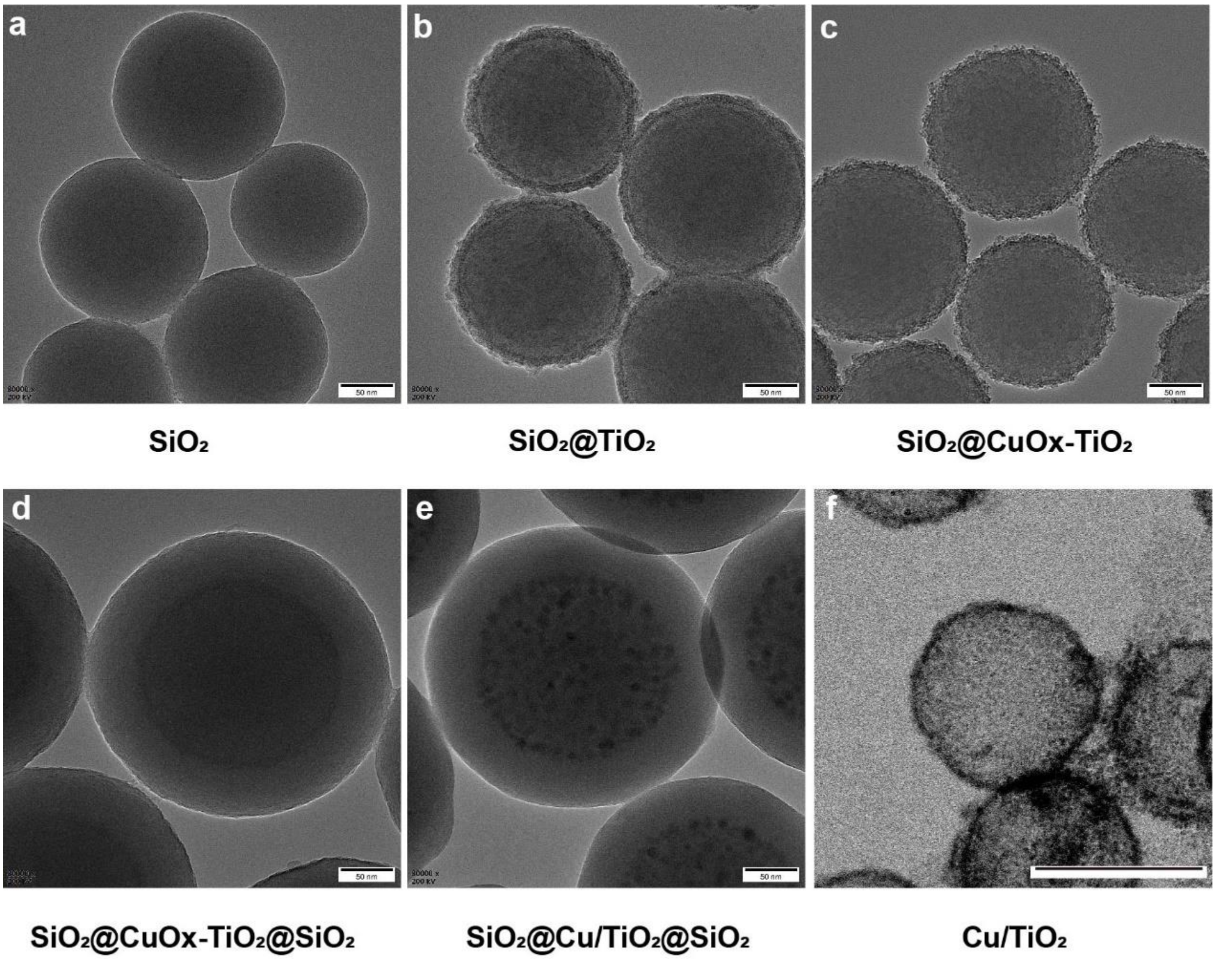

Figure S1. Synthesis process of single-atom Cu/TiO_2 nanosonosensitizers (scale bar: a~e: 50 nm, f: 200nm).

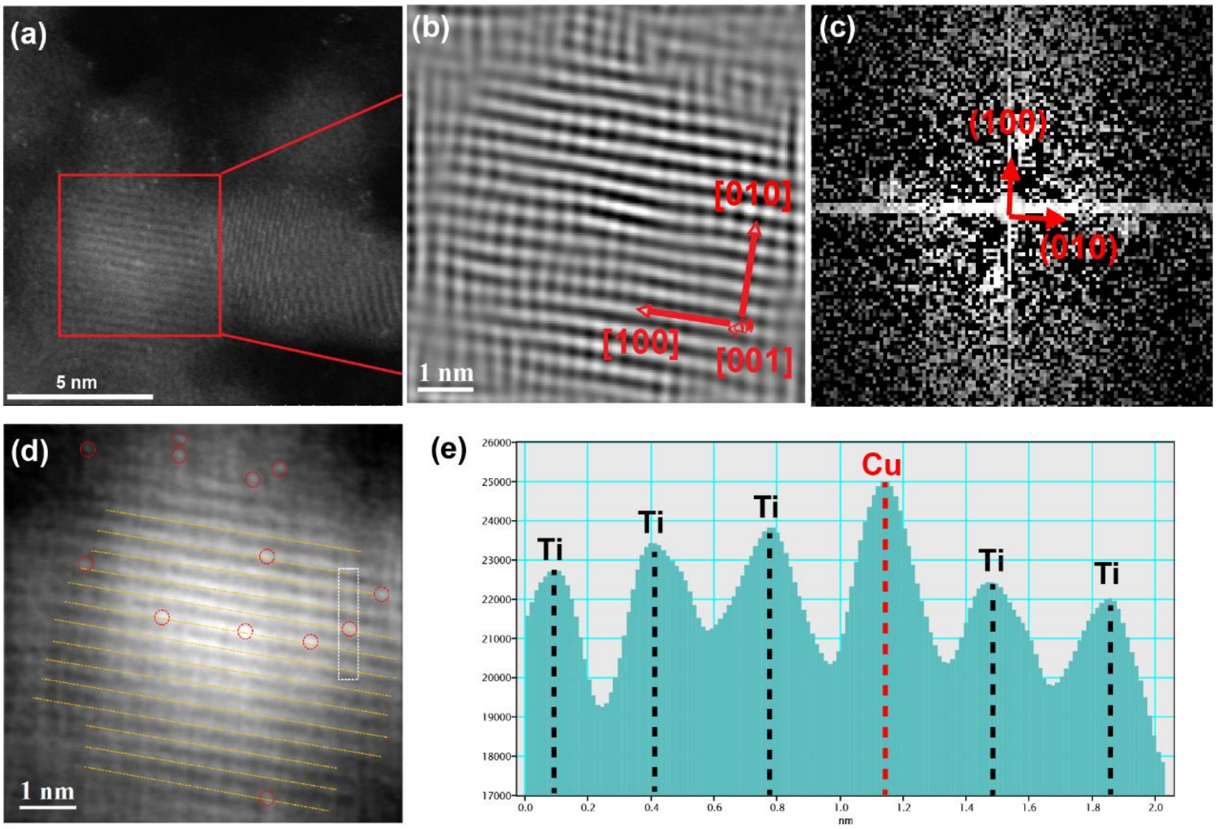

Figure S2. Atomic-level characterization of Cu/TiO_2 in the [100] direction. (a) Simulated HAADF-STEM and (b) HRTEM images of single-atom Cu/TiO_2 nanosonosensitizers. (c) Corresponding fast Fourier transform pattern. (d) Cs corrected HAADF STEM raw image of Cu/TiO_2. Red dotted circles mark isolated Cu atoms and yellow dotted lines represent atomic rows of Ti, respectively. (e) x–y line scan profile, measured from (d).

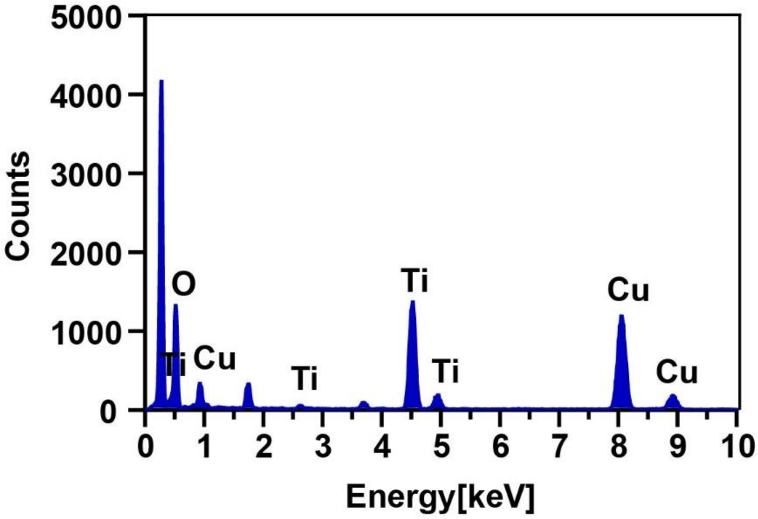

Figure S3. Corresponding EDS spectrum of Cu/TiO_2.

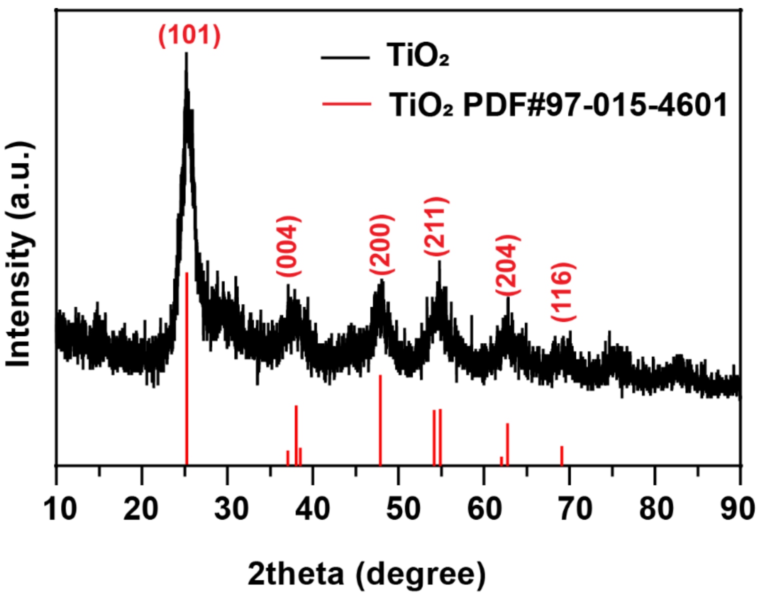

Figure S4. XRD pattern of TiO_2 nanosonosensitizers.

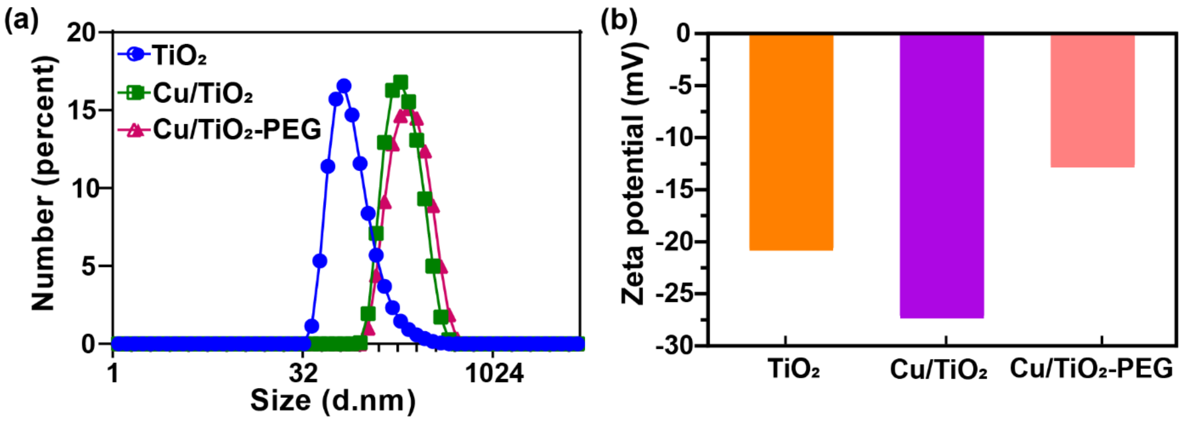

Figure S5. Size distribution (a) and zeta potential (b) of TiO_2, Cu/TiO_2, and Cu/TiO_2-PEG nanoparticles.

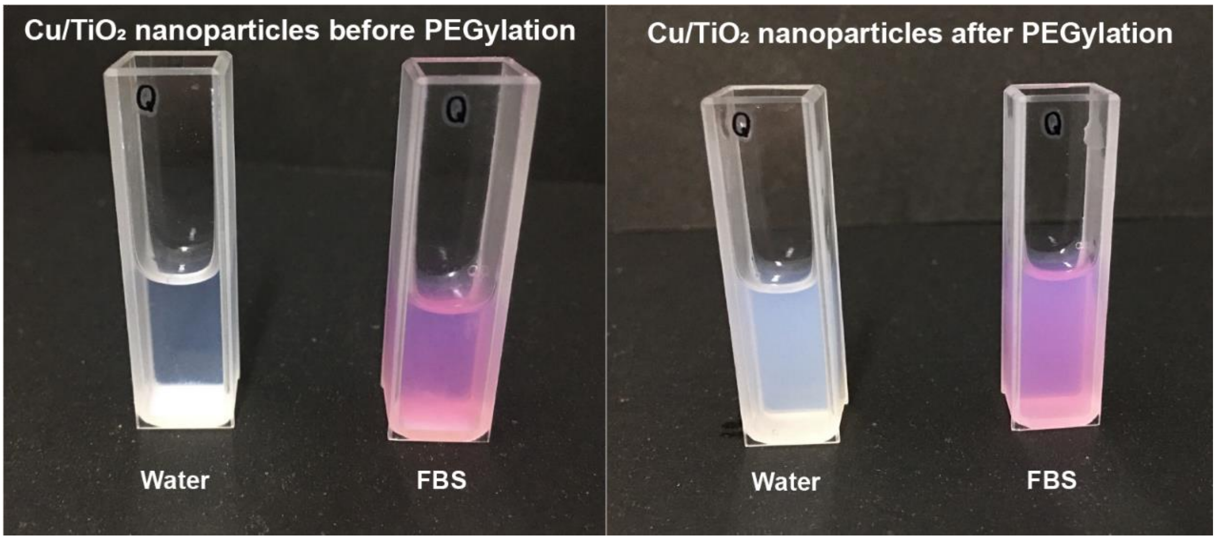

Figure S6. (a) Digital images of Cu/TiO_2 nanoparticles with or without NH_2-mPEG$_{2000}$ modification dispersed in different solutions (water and FBS) after 5 days.

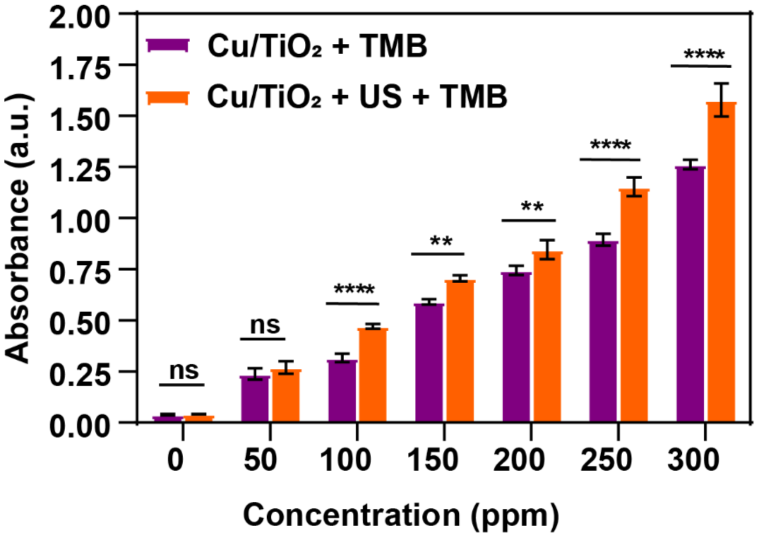

Figure S7. UV-vis absorbance of TMB to evaluate the ·OH generation of Cu/TiO_2 with or without US irradiation at different concentrations (0, 50, 100, 150, 200, 250, and 300 ppm). **P*< 0.05, ***P*< 0.01, ****P*<0.001, *****P*<0.0001 and ns for non-significant.

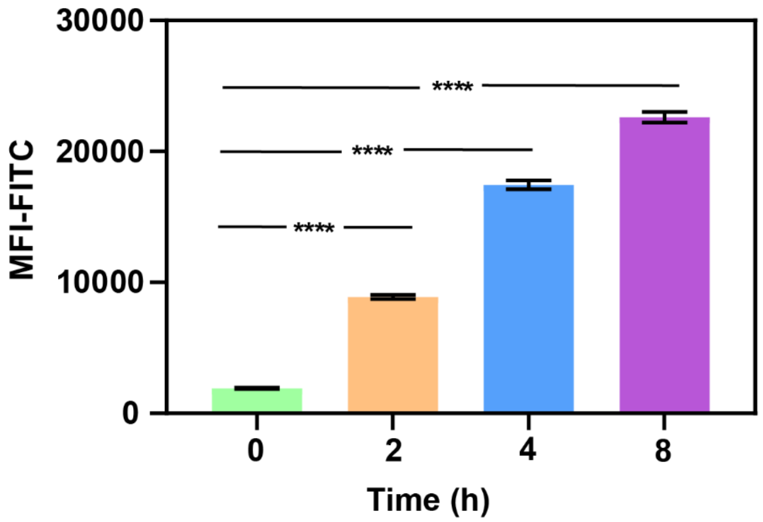

Figure S8. Average fluorescence intensity as measured by flow cytometry to analyze the intracellular uptake of FITC-labeled Cu/TiO_2-PEG after different intervals of co-incubation (0, 2, 4, and 8 h). *P< 0.05, **P< 0.01, ***P<0.001, ****P<0.0001 and ns for non-significant.

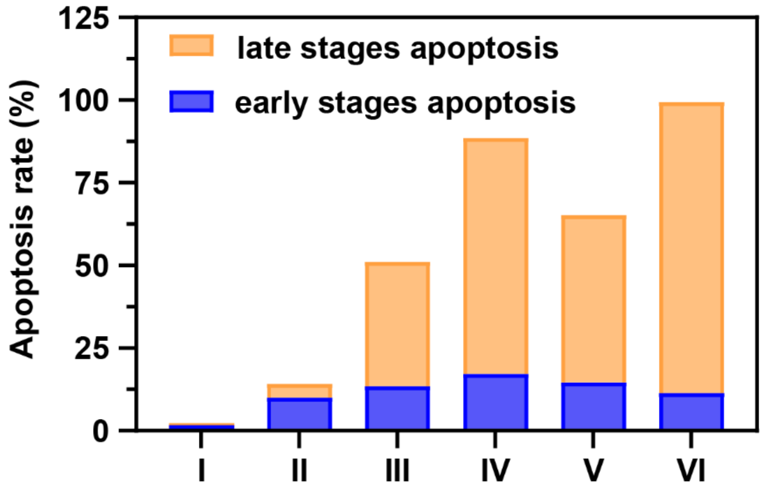

Figure S9. The apoptotic rate of 4T1 cancer cells after different treatments followed by staining with Annexin V-FITC and PI.

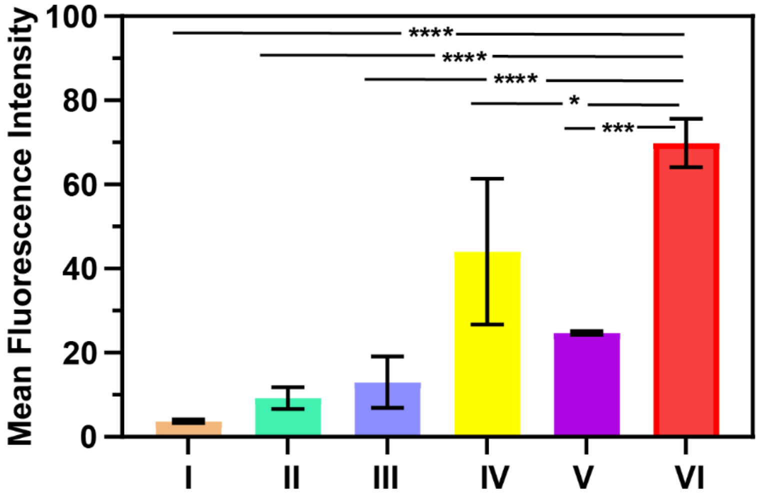

Figure S10. The mean fluorescence intensity of DCFH-DA-stained, US-irradiated 4T1 cells after different treatments (n=3). (I: control, II: only US, III: TiO_2-PEG, IV: TiO_2-PEG + US, V: Cu/TiO_2-PEG, VI: Cu/TiO_2-PEG + US). *P< 0.05, **P< 0.01, ***P<0.001, ****P<0.0001 and ns for non-significant.

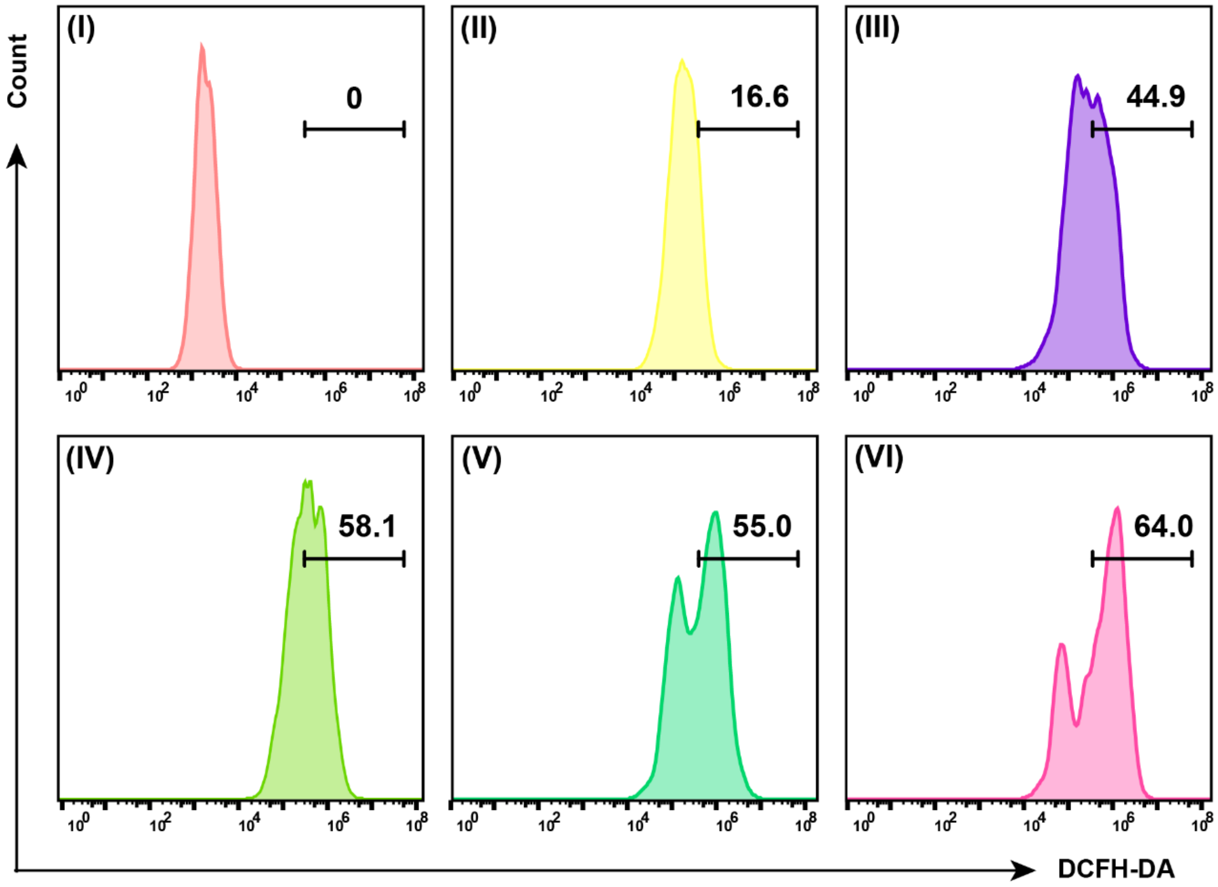

Figure S11. Flow cytometry of ROS generation as stained by DCFH-DA after different treatments. (I: control, II: only US, III: TiO_2-PEG, IV: TiO_2-PEG + US, V: Cu/TiO_2-PEG, VI: Cu/TiO_2-PEG + US).

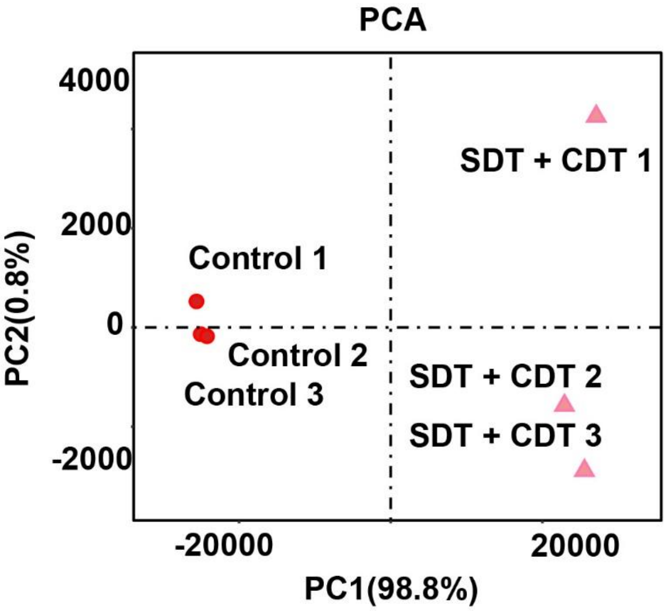

Figure S12. Principal component analysis (PCA) based on differentially expressed genes between the control group (Control) and Cu/TiO_2-PEG + US group (SDT + CDT).

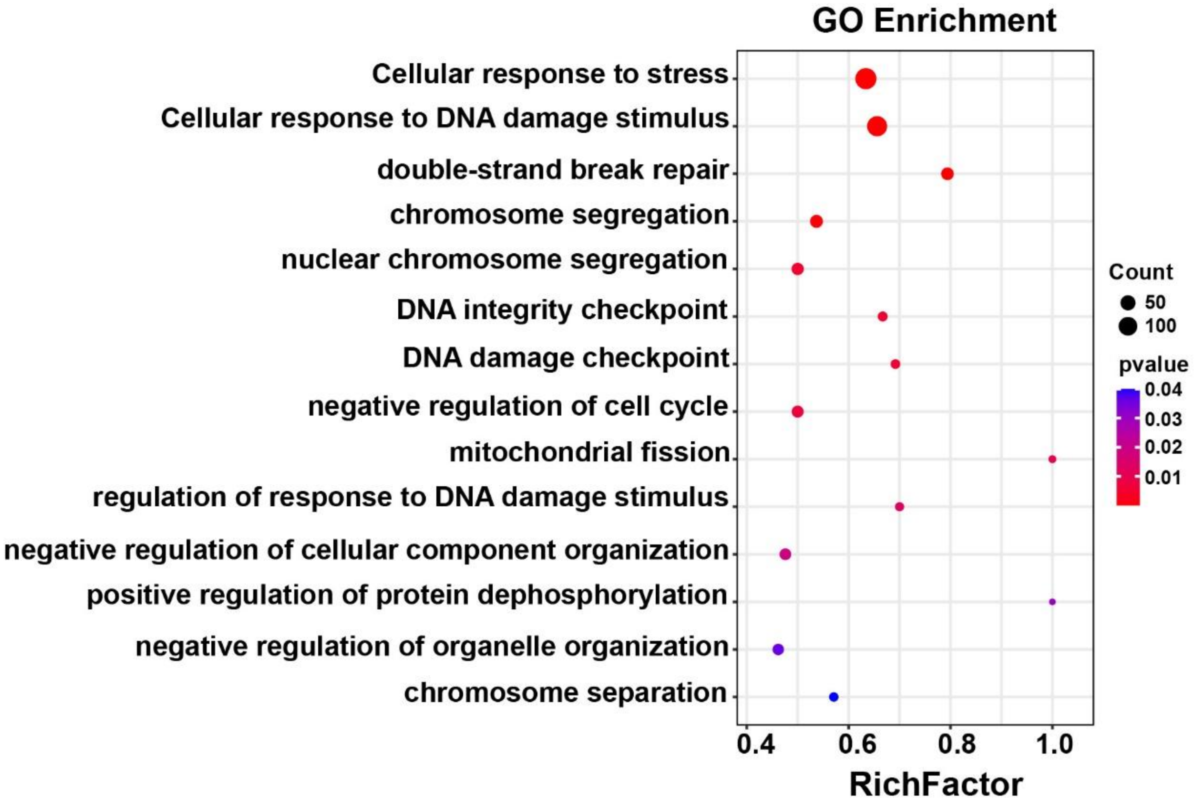

Figure S13. Bubble diagram of differential gene expression in GO analysis.

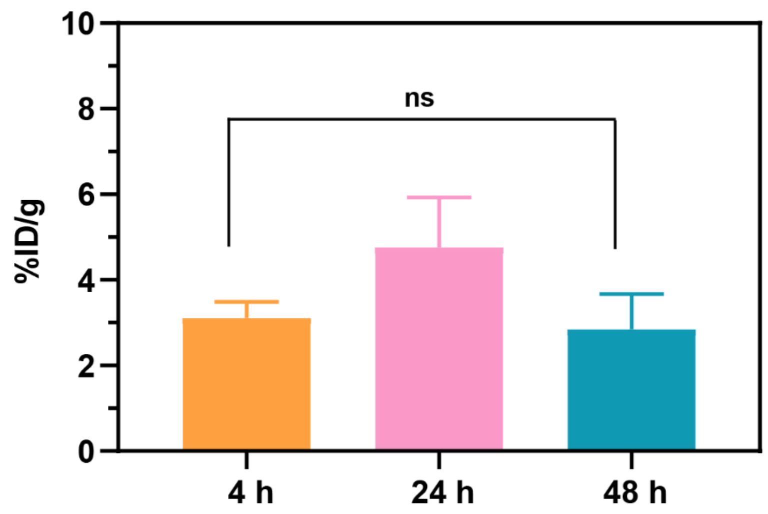

Figure S14. Quantitative analysis of Ti content in tumor at 4, 24 and 48 h post administration (n=3). *P< 0.05, **P< 0.01, ***P<0.001, ****P<0.0001 and ns for non-significant.

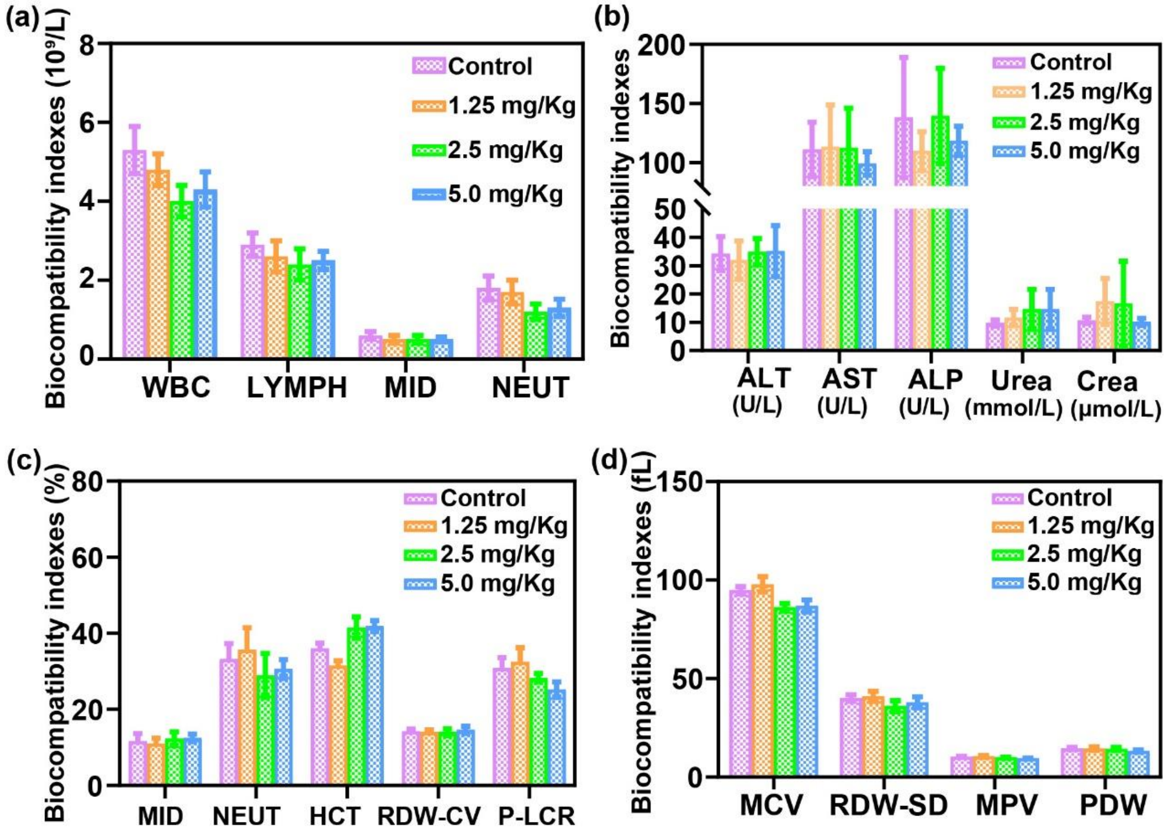

Figure S15. Relative blood indexes of mice from each group at different Cu/TiO_2-PEG doses of the control group, 1.25 mg kg^{-1}, 2.5 mg $kg^{-1,}$ and 5 mg kg^{-1} at the 30th post-injection (WBC: white blood cell count, LYMPH: lymphocyte number, MID: Intermediate cell, NEUT: neutrophil, ALT: alanine aminotransferase, AST: aspartate aminotransferase, ALP: alkaline phosphatase, Urea: urea, Crea: creatinine, MID%: Intermediate cell ratio, NEUT%: neutrophil ratio, HCT: blood routine levels of hematocrit, RDW-CV: Red blood cell volume distribution width-CV, P-LCR: platelet -larger cell ratio, MCV: mean corpuscular volume, RDW-SD: Red blood cell volume distribution width-SD, MPV: mean platelet volume, PDW: platelet distribution width).

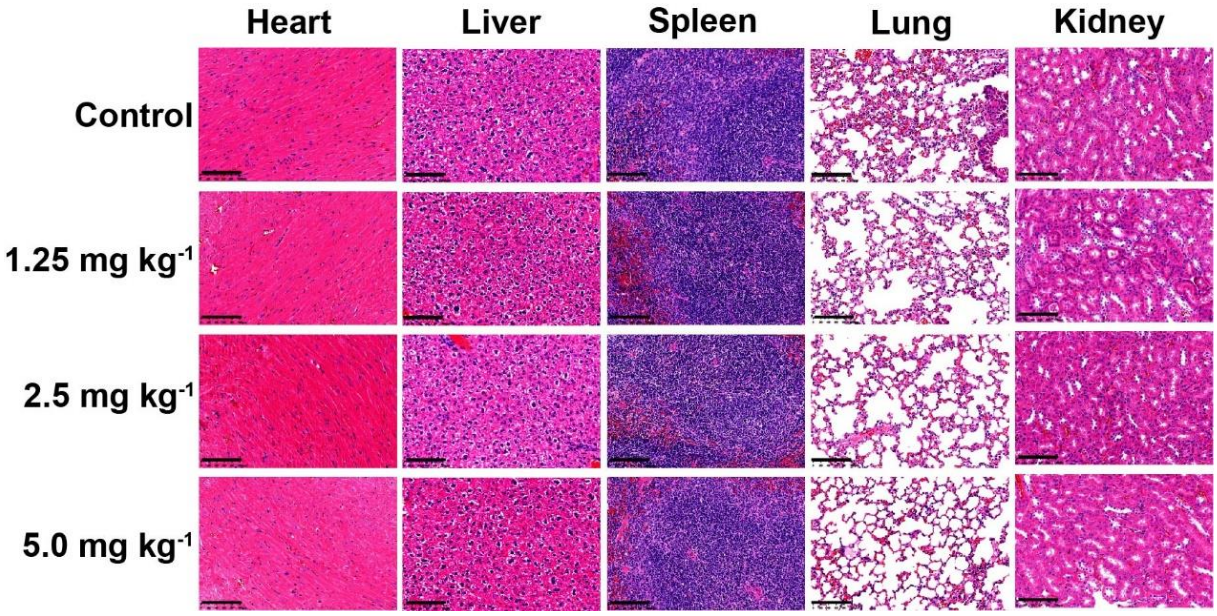

Figure S16. H&E staining of major organs, including heart, liver, spleen, lung, and kidney at the 30th post-injection of Cu/TiO_2-PEG at elevated doses. Scale bar: 100 μm.

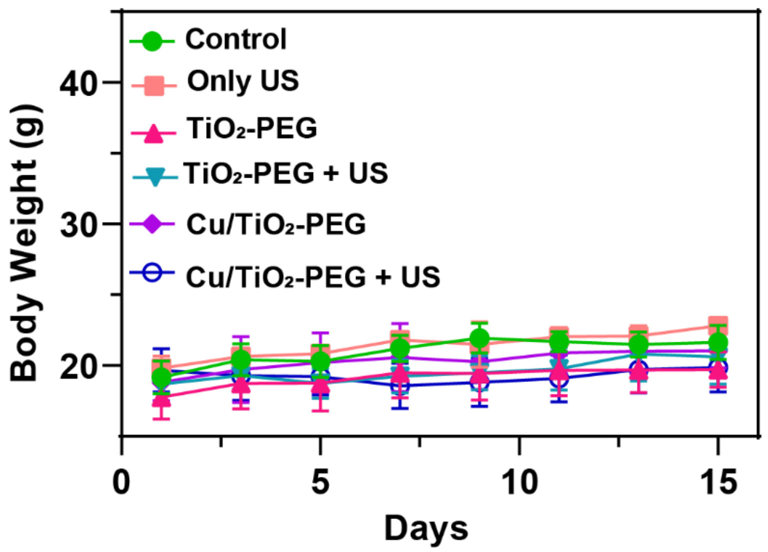

Figure S17. Time-dependent body-weight curves in each group.

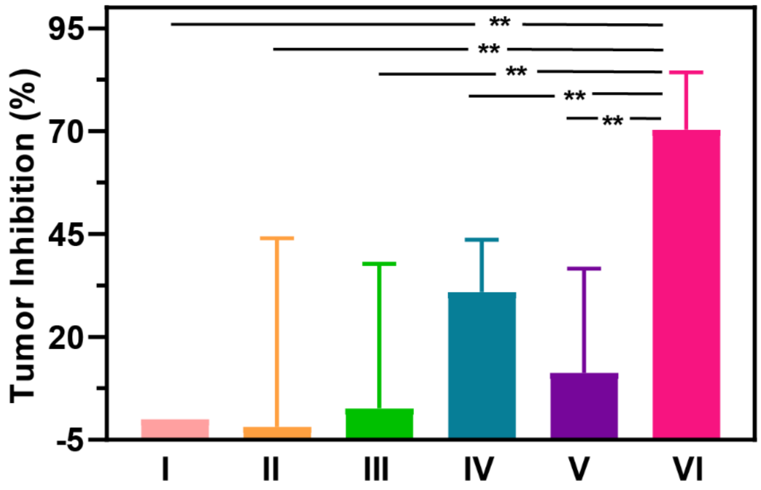

Figure S18. Tumor inhibition ratio of experimental groups. Significant differences appeared between the Cu/TiO_2-PEG + US group and the other experimental groups. (I: control, II: only US, III: TiO_2-PEG, IV: TiO_2-PEG + US, V: Cu/TiO_2-PEG, VI: Cu/TiO_2-PEG + US). *P< 0.05, **P< 0.01, ****P<0.0001 and ns for non-significant.

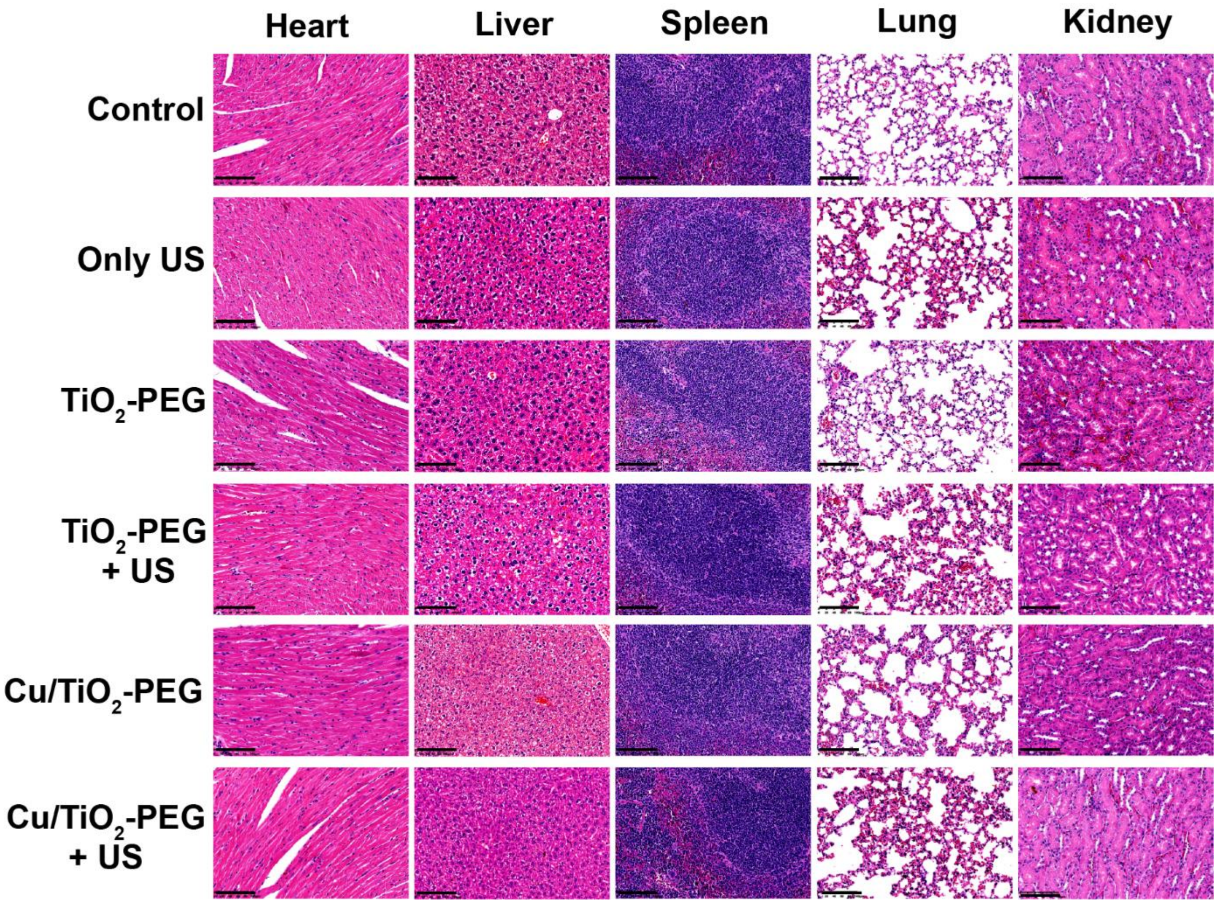

Figure S19. H&E staining of the major organs (heart, liver, spleen, lung and kidney) of 4T1 tumor-bearing mice after various treatments. Scale bar: 100 μm.

Reference

[1] B. Lee, S. Park, M. Kim, A. Sinha, S. Lee, E. Jung, W. Chang, K. Lee, J. Kim, S. Cho, H. Kim, K. Nam, T. Hyeon, *Nat Mater*. **2019**, 18, 620.

[2] a) J. Perdew, Y. Wang, *Phys Rev B Condens Matter*. 1992, 45,13244; b) J. Perdew, K. Burke, M. Ernzerhof, *Phy Rev let*. **1996**, *77*, 3865.

[3] G. Kresse, J. Furthmüller, *Phys Rev B Condens Matter*. **1996**, 54, 11169.

[4] a) S. Grimme, J. Antony, S. Ehrlich, H. Krieg, *J Chem Phys*. **2010**, *132*, 154104; b)S. Grimme; S. Ehrlich; L. Goerigk, *J Comput Chem*. **2011**, *32*, 1456.
